# High Intensity Interval Training (HIIT) as a Potential Countermeasure for Phenotypic Characteristics of Sarcopenia: A Scoping Review

**DOI:** 10.3389/fphys.2021.715044

**Published:** 2021-08-24

**Authors:** Lawrence D. Hayes, Bradley T. Elliott, Zerbu Yasar, Theodoros M. Bampouras, Nicholas F. Sculthorpe, Nilihan E. M. Sanal-Hayes, Christopher Hurst

**Affiliations:** ^1^School of Health and Life Sciences, University of the West of Scotland, Hamilton, United Kingdom; ^2^Translational Physiology Research Group, School of Life Sciences, University of Westminster, London, United Kingdom; ^3^Active Ageing Research Group, Institute of Health, University of Cumbria, Lancaster, United Kingdom; ^4^Lancaster Medical School, Lancaster University, Lancaster, United Kingdom; ^5^The Centre for Ageing Research, Lancaster University, Lancaster, United Kingdom; ^6^Department of Psychology, Lancaster University, Lancaster, United Kingdom; ^7^AGE Research Group, Translational and Clinical Research Institute, Newcastle University, Newcastle upon Tyne, United Kingdom; ^8^National Institute for Health Research Newcastle Biomedical Research Centre, Newcastle upon Tyne Hospitals National Health Service Foundation Trust and Newcastle University, Newcastle upon Tyne, United Kingdom

**Keywords:** aging, exercise, HIIT, high intensity, power, sarcopenia, sprint, strength

## Abstract

**Background:** Sarcopenia is defined as a progressive and generalized loss of skeletal muscle quantity and function associated predominantly with aging. Physical activity appears the most promising intervention to attenuate sarcopenia, yet physical activity guidelines are rarely met. In recent years high intensity interval training (HIIT) has garnered interested in athletic populations, clinical populations, and general population alike. There is emerging evidence of the efficacy of HIIT in the young old (i.e. seventh decade of life), yet data concerning the oldest old (i.e., ninth decade of life onwards), and those diagnosed with sarcopenic are sparse.

**Objectives:** In this scoping review of the literature, we aggregated information regarding HIIT as a potential intervention to attenuate phenotypic characteristics of sarcopenia.

**Eligibility Criteria:** Original investigations concerning the impact of HIIT on muscle function, muscle quantity or quality, and physical performance in older individuals (mean age ≥60 years of age) were considered.

**Sources of Evidence:** Five electronic databases (Medline, EMBASE, Web of Science, Scopus, and the Cochrane Central Register of Controlled Trials [CENTRAL]) were searched.

**Methods:** A scoping review was conducted using the Arksey and O'Malley methodological framework (2005). Review selection and characterization were performed by two independent reviewers using pretested forms.

**Results:** Authors reviewed 1,063 titles and abstracts for inclusion with 74 selected for full text review. Thirty-two studies were analyzed. Twenty-seven studies had a mean participant age in the 60s, two in the 70s, and three in the 80s. There were 20 studies which examined the effect of HIIT on muscle function, 22 which examined muscle quantity, and 12 which examined physical performance. HIIT was generally effective in Improving muscle function and physical performance compared to non-exercised controls, moderate intensity continuous training, or pre-HIIT (study design-dependent), with more ambiguity concerning muscle quantity.

**Conclusions:** Most studies presented herein utilized outcome measures defined by the European Working Group on Sarcopenia in Older People (EWGSOP). However, there are too few studies investigating any form of HIIT in the oldest old (i.e., ≥80 years of age), or those already sarcopenic. Therefore, more intervention studies are needed in this population.

## Key Points

- A variety of intensity prescriptions were utilized in previous experiments, which included “all-out” effort, percentage of maximal heart rate, perceived a percentage of peak oxygen uptake, percentage of intensity at termination of a ramped exercise test, percentage of peak instantaneous power, rating of perceived exertion, and percentage of maximum gait speed.
- Twenty-seven studies had a mean participant age in the 60s, two in the 70s, and three in the 80s. There were 20 studies which examined the effect of HIIT on muscle function, 22 studies which examined the effect of HIIT on muscle quantity, and 12 studies which examined the effect of HIIT on physical function (which are the outcomes used to diagnose sarcopenia).
- No previous investigation had considered HIIT in a sarcopenic or pre-sarcopenic population, and only three studies were in the oldest old humans.

## Introduction

### Rationale

Sarcopenia is a progressive skeletal muscle disorder, characterized by reduced skeletal muscle quantity and function which is associated with a range of negative health outcomes including frailty, falls, reduced quality of life, and mortality (Cruz-Jentoft and Sayer, 2019; Cruz-Jentoft et al., 2019). In addition to these individual health impacts, sarcopenia places a considerable economic burden on healthcare systems with the associated costs in the UK estimated at £2.5 billion per year (Pinedo-Villanueva et al., 2019). Taken together, these effects highlight the need to develop treatment strategies to counteract the deleterious consequences of sarcopenia.

Factors including chronic inflammation, mitochondrial dysfunction, and reduced satellite cell function contribute to the onset and progression of sarcopenia (Ziaaldini et al., 2017). Exercise training has the potential to counteract these cellular, molecular, and neural alterations (Marzetti et al., 2017; Seo and Hwang, 2020) with aerobic and resistance exercise capable of inducing differential adaptations (Hawley et al., 2014). Previous work has demonstrated that resistance exercise has multisystem effects, acting at both the physiological [e.g., improvements in mitochondrial function (Melov et al., 2007) and reduced inflammation (Beyer et al., 2012)] and the functional level [e.g., improvements in muscle strength and physical performance (Peterson et al., 2010; Steib et al., 2010)]. To date there remains no pharmacological treatment approved for the treatment of sarcopenia and resistance exercise training is recommended as its primary treatment (Dent et al., 2018). Given the multi-factorial nature of sarcopenia, exercise programmes for older adults living with sarcopenia often involve a combination of exercise modes (Witham et al., 2020) with the aim of simultaneously improving muscular and cardiorespiratory function (Hurst et al., 2019a). Offering a range of alternative exercise training approaches which can simultaneously improve multiple outcomes (e.g., muscle strength, physical performance, and cardiorespiratory fitness) could help to maximize the potential of exercise as a therapeutic strategy for older people living with sarcopenia.

High intensity interval training (HIIT) has previously been shown to exert substantial cardio-protective effects, across a range of population groups (Knowles et al., 2015; Hwang et al., 2016; Batacan et al., 2017; Füzéki and Banzer, 2018; Hannan et al., 2018; Hayes et al., 2020; Herbert et al., 2021). In the clinical context, HIIT has been shown to be a safe, feasible and effective therapeutic strategy in patients living with diabetes (Little et al., 2011), heart failure (Angadi et al., 2015) and coronary artery disease (Warburton et al., 2005). From a pragmatic perspective, HIIT can be embedded within the clinical pathway (Way et al., 2020) and can be delivered using a range of exercise modes (e.g., stair climbing, stepping, cycling, walking).

Despite this, much less is known about how HIIT could improve elements of muscular structure and function. A recent narrative review (Callahan et al., 2021) outlined several mechanistic explanations as to why HIIT *might* be anabolic in nature. These authors called for further investigation of HIIT in populations of different age groups and training status to explore this phenomenon further. Moreover, they proposed HIIT may be beneficial in middle and older age where physical conditioning (i.e., aerobic fitness) and increased muscle quantity were simultaneously desired. Whether HIIT could provide the necessary improvements in muscle quantity, quality, and strength, in addition to cardioprotective effects however, remain unclear (Hurst et al., 2019b). The potential for HIIT to simultaneously induce improvements in cardiometabolic health and muscular health is an appealing strategy. However, until now there has not been a comprehensive review of HIIT within older adults pertaining to phenotypic characteristics of sarcopenia using a systematic search strategy.

Given that exercise programmes delivered to older people with sarcopenia in clinical practice are varied and often poorly prescribed (Witham et al., 2020), delivering effective and engaging exercise programmes to older people is of prime concern (Dismore et al., 2020; Collado-Mateo et al., 2021). HIIT is reportedly enjoyable (Thum et al., 2017), can be completed without gym equipment (Blackwell et al., 2017; Dunford et al., 2021; Yasar et al., 2021), and deliver self-perceived health and fitness improvements (Knowles et al., 2015). However, before HIIT can be proposed as a viable countermeasure to phenotypic characteristics of sarcopenia, it is important to consider the existing literature in terms of methodologies, quality of research and heterogeneity, to determine whether a systematic review and meta-analysis is possible, and if not to identify the areas in which the current literature is deficient. A comprehensive review of HIIT and its effect on phenotypic characteristics of sarcopenia is important for clinicians and exercise practitioners to ensure they are equipped to support community-dwelling older adults and their families/caregivers. Therefore, it seemed prudent to conduct a scoping review in this area to map the existing literature in terms of the volume, nature, and characteristics of the primary research (Arksey and O'Malley, 2005). We used a scoping review rather than systematic review and meta-analysis because our aim was not to ask a precise question and were more interested in the characteristics of investigations conducted (Munn et al., 2018). Moreover, the topic has not yet been extensively reviewed and may have been complex or heterogeneous in nature. If existing research was heterogeneous, a systematic review and meta-analysis would not have been possible, and therefore we opted to scope the area in this manuscript (Mays et al., 2001).

### Objectives

We aimed to provide an overview of existing literature relating to phenotypic characteristics of sarcopenia pre- and post-HIIT in older adults. The four specific objectives of this scoping review were to (1) conduct a systematic search of the published literature for the effect of HIIT on muscle strength, muscle quantity or quality, and physical performance [aligned to the 2018 operational definition of sarcopenia (Cruz-Jentoft et al., 2019)] in older adults, (2) map characteristics and methodologies used and classified as “HIIT” within the interventions, (3) outline the range and characteristics of outcome variables used, and (4) provide recommendations for the advancement of the investigative area.

## Methods

### Protocol and Registration

The review was conducted and reported according to the Preferred Reporting Items for Systematic Reviews and Meta-Analyses extension for scoping reviews (PRISMA-ScR) guidelines (Tricco et al., 2018) and the five-stage framework outlined in Arksey and O'Malley (Arksey and O'Malley, 2005). A review protocol was not published.

### Eligibility Criteria

Studies that met the following criteria were included: (1) involvement of human participants with a mean age of ≥ 60 years [considered the start of old age (United Nations, 2020)]; (2) not a review; (3) an intervention which included bouts of high intensity exercise interspersed with periods of recovery, including exercise defined as HIIT or sprint interval training (SIT). We defined high intensity as exercise >85% peak oxygen uptake (VO_2peak_) or 85% maximal heart rate (HR_max_) or equivalent perception-based approaches (e.g., Borg 6–20 scale or similar); (4) employing an intervention design and include an exercise training period of >2 weeks; (5) including HIIT in isolation or performed in combination with another form of exercise; (6) including outcome measures related to either (i) muscle function (either strength or power), (ii) muscle quantity, or (iii) physical performance.

### Search Strategy

The search strategy consisted of a combination of free-text and MeSH terms relating to “high-intensity interval training,” “sarcopenia,” and “older adults” which were developed through examination of previously published original and review articles (e.g., screening of titles, abstracts, keywords). Filters were applied to ensure that only records published in English language involving human participants were included in the search results. Full search terms and the complete search strategy can be found in the online Supplementary Material associated with this article (Supplementary Material 1).

### Information Sources

Five electronic databases (Medline, EMBASE, Web of Science, Scopus, and the Cochrane Central Register of Controlled Trials [CENTRAL]) were searched to identify original research articles published from the earliest available up until 12th March 2020. Reference lists from included studies and previously published review articles were examined for potentially eligible papers.

### Study Selection

Data were extracted by two reviewers (LH & CH) independently and compared in an unblinded and standardized manner. Once each database search was completed and manuscripts were sourced, all studies were downloaded into a single reference list with duplicates removed. Titles and abstracts were then screened for eligibility and full texts were only retrieved for studies with HIIT incorporated. Two reviewers then read and coded all the included articles using the PEDro scale (de Morton, 2009). Full texts were then thoroughly assessed using the complete eligibility criteria with first (LH) and last (CH) authors confirming inclusion and exclusion. Following this quality assessment, the same reviewers read and coded each of the studies and assessed the following moderators: design method (randomized control trial; RCT, controlled trial; CT or uncontrolled trial; UCT), combined or HIIT in isolation, and outcome variable. Furthermore, participant descriptions and training programme variables were extracted with as much detail provided by the authors. Any disagreement between reviewers was discussed in a consensus meeting, and unresolved items were addressed by a third reviewer.

### Data Items

Data extracted from each study included sample size, group descriptions, study design, analysis method, and outcome data. Methodological quality was assessed using the modified 0–10 PEDro scale (de Morton, 2009). The primary outcome variables were defined as muscle strength or power, muscle quantity or quality, and physical performance, pre- and post-intervention. There was heterogeneity in study inclusion criteria, interventions, assessment tools, and outcomes, thus a pooled analysis was not appropriate.

## Results

### Study Selection

**Figure 1:**
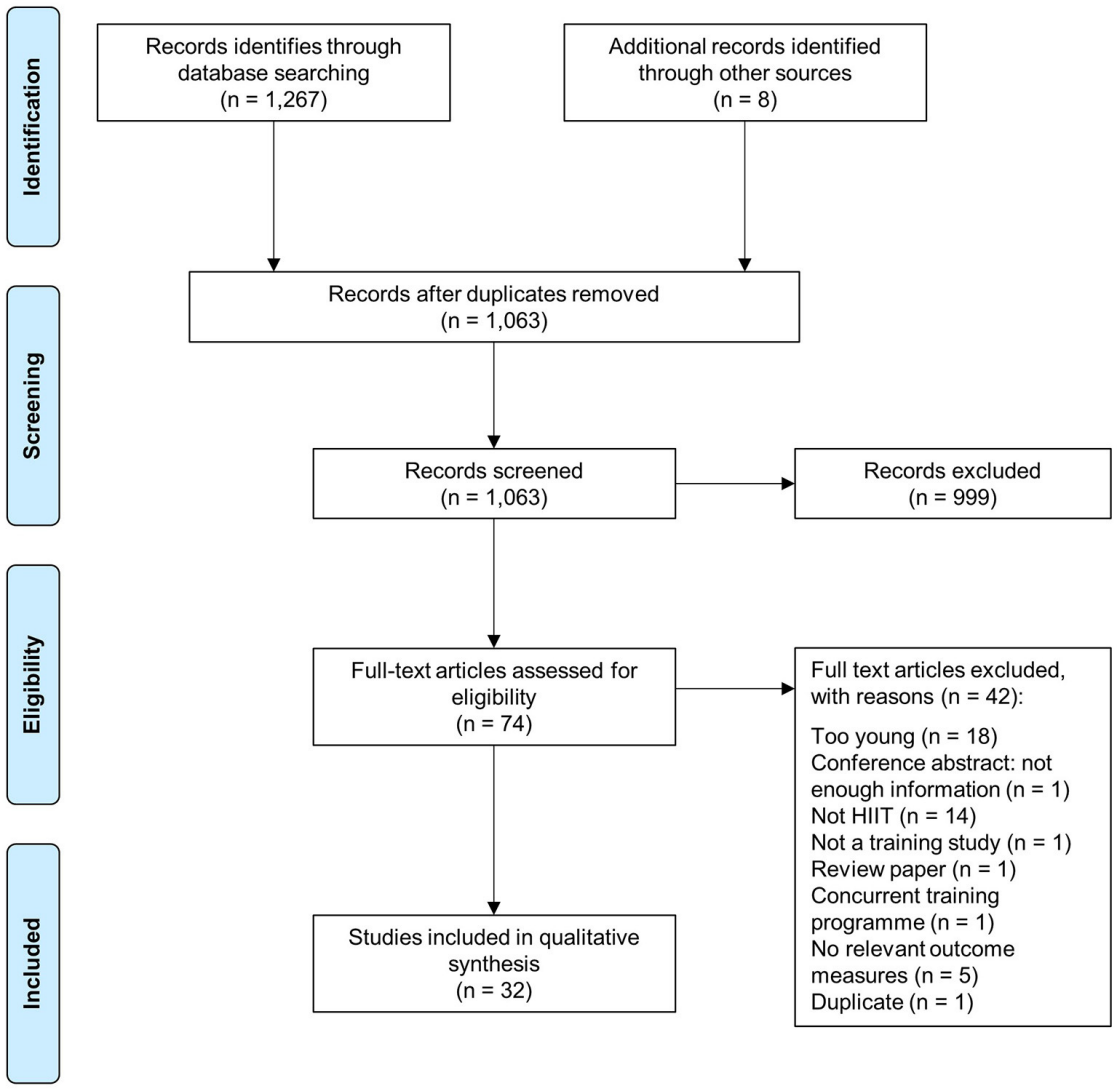
Schematic flow diagram describing exclusions of potential studies and final number of studies.

Following the initial database search, 1,267 records were identified (Figure 1). Once duplicates were removed, 1,063 titles and abstracts remained, and were screened for inclusion, resulting in 74 full-text articles being screened. Of these, 42 were excluded and 32 remained.

### Study Characteristics

**Figure 2:**
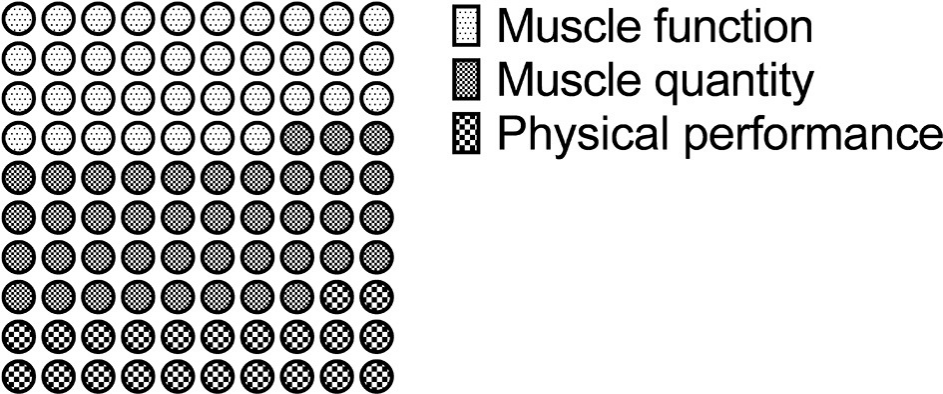
Schematic representation of frequency of outcome examined (*n* = 54) within the 32 included studies concerning HIIT and phenotypic characteristics of sarcopenia.

Of the 32 studies included, 14 were RCTs (Adamson et al., 2014, 2020; Hwang et al., 2016; Coetsee and Terblanche, 2017; Sculthorpe et al., 2017; Aboarrage Junior et al., 2018; Malin et al., 2018; Martins et al., 2018; Ballesta-García et al., 2019; Hurst et al., 2019c; Jiménez-García et al., 2019; Nunes et al., 2019; Taylor et al., 2019; Coswig et al., 2020), one was a quasi-experimental, non-randomized, single-blinded controlled study (Losa-Reyna et al., 2019), 16 were observational cohort studies (Bruseghini et al., 2015, 2019; Boereboom et al., 2016; Guadalupe-Grau et al., 2017; Hayes et al., 2017; Herbert et al., 2017a,b; Robinson et al., 2017; Wyckelsma et al., 2017; Andonian et al., 2018; Bartlett et al., 2018; Buckinx et al., 2018, 2019; Søgaard et al., 2018, 2019; Snijders et al., 2019), and one was a pilot study (although randomized; (Beetham et al., 2019) (Table 1). Where a study had multiple outcome measures, they were examined separately. Three out of 32 (9%) included HIIT in a multicomponent intervention (Guadalupe-Grau et al., 2017; Losa-Reyna et al., 2019; Snijders et al., 2019). Sixteen studies included HIIT on a cycle ergometer, six included HIIT on a treadmill, seven included resistance exercise HIIT (including bodyweight exercises), two included HIIT on an elliptical trainer, and one study did not detail the intervention. Three studies used an “all-out” intensity, 15 used a percentage of HR_max_ or heart rate reserve (HRR) to prescribe intensity, four used a percentage of VO_2peak_ to prescribe intensity, three used a percentage of intensity at termination of a ramped incremental exercise protocol to prescribe intensity, four used percentage of peak power output to prescribe intensity, one used the Borg scale to prescribe intensity, one study used a percentage of maximum gait speed to prescribe intensity, and one study did not detail the intervention. Twenty-seven studies had a mean age in the 60s, two in the 70s, and three in the 80s. One study considered frail participants. There were 20 studies which examined the effect of HIIT on muscle function, 22 studies which examined the effect of HIIT on muscle quantity, and 12 studies which examined the effect of HIIT on physical function (Figure 2). Several studies investigated more than one parameter, thus why the sum of the studies above is greater than the number of included studies.

**Table 1:** General study information of investigations concerning HIIT and phenotypic characteristics of sarcopenia.

**Reference**	**Population**	**Study design**	**Study protocol available/** **Preregistered**	**Intervention characteristics**						**Outcome(s)**	**PEDro score**
				**Duration (weeks)**	**Total sessions**	**Exercise protocol**	**Exercise intensity**	**Adherence/** **Compliance/** **Attendance**	**Adverse events**		
Aboarrage Junior et al. (2018)	25 untrained females total, 15 in training group (aged 65 ± 7 years); normal body mass; disease free.	RCT	No	24 weeks	72	5 min warm-up preceded jump-based SIT (20 min of 20 repetitions of 30 s work, 30 s rest); 5 min cool-down on a cycle ergometer.	All-out	>90% inclusion criteria	“No participants in either group left the study or Presented any injuries as result of the exercise program”	Muscle quantity Physical performance	5
Adamson et al. (2014)	12 untrained older adults in total, 6 in training group (aged 65 ± 4 years); normal body mass; disease free.	RCT	No	6 weeks	12	6–10 6 s sprints on a cycle ergometer against ~7% body mass,~ 60 s rest.	All-out	-	Not reported	Physical performance	5
Adamson et al. (2020)	34 untrained older adults, 11 in once per week training, and 11 in twice per week training group (aged 65 ± 3 years); disease free.	RCT	No	8 weeks	8 for the once per week training group 16 for the twice per week training group	6–10 6 s sprints on a cycle ergometer against ~7% body mass,~ 60 s rest.	All-out	-	Not reported	Physical performance	5
Andonian et al. (2018)	21 untrained, sedentary older adults with rheumatoid arthritis (*n* = 12; 64 ± 7 years) or prediabetes (*n* = 9; 71 ± 5 years), free of CVD or diabetes, able-bodied.	Observational cohort study	The study was registered with ClinicalTrials.gov	10 weeks	30	5 min warm-up preceded 90 s work, 90 s rest); 5 minute cool-down on a treadmill.	80-90% HRR	-	Not reported	Muscle quantity	2
Ballesta-García et al. (2019)	54 individuals (*n* = 18, 66 ± 5 years in the HIIT group, *n* = 18, 70 ± 9 years in the MICT group, and, *n* = 18, 67 ±69 years in the control group group), without hypertension or a disease that would interfere with exercise.	RCT with MICT and non-exercise control	The study was registered prospectively with ClinicalTrials.gov	18 weeks	36	1–1.5 min work, 2–2.5 min rest). 6–12 intervals. The programme was progressed over the 18 weeks. “Movements of the lower limbs, combined with the movements of the upper limbs with or without external load.”	14–18 on the Borg scale	>80% inclusion criteria. There were registered adverse events in MICT and control groups. Four women in the MICT group and one in control were lost to follow-up due to eye surgery, foot surgery, clavicle fracture, and two hip fractures after a fall. These adverse events did not occur during exercise classes.	Not reported	Physical performance Muscle function	6
Bartlett et al. (2018)	12 untrained, sedentary older adults with rheumatoid arthritis (64 ± 7 years), free of CVD or diabetes, able-bodied.	Observational cohort study	The study was registered with ClinicalTrials.gov	10 weeks	30	5 min warm-up preceded 60–90 s work, 60–90 s rest; 5 min cool-down on a treadmill. Time per session was matched at 30 min.	80–90% VO_2_ reserve targeted. 85 ± 5% achieved.	99% adherence.	Not reported	Physical performance	2
Beetham et al. (2019)	21 individuals with stage 3–4 kidney disease (*n* = 9, 61 ± 6 years in the HIIT group and *n* = 5, 63 ± 11 years in the MICT group), overweight and varied diabetic status.	Randomized pilot trial vs MICT	The study was registered at the Australian and New Zealand Clinical Trials Registry	12 weeks	36	5 min warm-up preceded 4 × 4 min intervals with 3 min rest on a treadmill. The programme was progressed over the 12 weeks.	80–95% peak heart rate.	33/36 for HIIT, 34/36 for MICT.	None attributed to the intervention.	Muscle quantity	8
Boereboom et al. (2016)	21 individuals (aged ~ 67 years)	Observational cohort study	The study was registered with ClinicalTrials.gov	31 days	12	2 min warm-up preceded 5 × 60 s intervals with 90 s rest on a cycle ergometer.	100–110% power achieved during a ramped CPET protocol to failure.	12 (full compliance)	Not reported	Muscle quantity	2
Bruseghini et al. (2015)	12 healthy older adults (aged 68 ± 4 years).	Proof-of-concept observational cohort study	No	8 weeks	24	10 min warm-up preceded 7 × 2 min intervals with 2 min rest on a cycle ergometer.	85–95% VO_2peak_	Not reported	Not reported	Muscle function Muscle quantity	2
Bruseghini et al. (2019)	12 moderately active healthy men (aged 69 ± 4 years), normal body mass, disease free.	Observational cohort study	No	8 weeks	24	10 min warm-up preceded 7 × 2 min intervals with 2 min rest on a cycle ergometer. The programme was progressed every 2 weeks.	85–95% VO_2peak_	Not reported	None attributed to the intervention.	Muscle function	2
Buckinx et al. (2019)	33 untrained adults (aged 69 ± 4 years), non-smoking, low alcohol consuming, postmenopausal (if female), without counter-indication to exercise.	Observational cohort dataset	No	12 weeks	36	5 min warm-up preceded 10 × 30 s intervals with 90 s rest on an elliptical device. The programme was progressed.	80–85% peak heart rate or >17 on the Borg scale	>80% inclusion criteria	Not reported	Physical performance Muscle function Muscle quantity	3
Buckinx et al. (2018)	30 untrained adults (aged 69 ± 4 years), non-smoking, low alcohol consuming, postmenopausal (if female), without counter-indication to exercise.	Observational cohort dataset	No	12 weeks	36	5 min warm-up preceded 10 × 30 s intervals with 90 s rest on an elliptical device. The programme was progressed.	80–85% peak heart rate or >17 on the Borg scale	>80% inclusion criteria	Not reported	Physical performance	3
Coetsee and Terblanche (2017)	67 inactive individuals (*n* = 13, 65 ± 6 years in the HIIT group and *n* = 129, 63 ± 6 years in the control group), normal BMI, no cognitive impairment, and no comorbidities.	RCT	No	16 weeks	48	4 × 4 min intervals with 3 min rest on a treadmill. The programme was progressed	90–95% peak heart rate.	Not reported	Not reported	Physical performance	5
Coswig et al. (2020)	46 untrained female nursing home residents (aged 81 ± 5 years), *n* = 15 in HIIT group. Comorbidities that did not preclude involvement.	RCT with MICT as a positive control group	No	8 weeks	16	5 min warm-up preceded 4 × 4 min intervals with 4 min rest on a treadmill. The programme was progressed	85–95% peak heart rate.	>80% inclusion criteria.	Not reported	Physical performance Muscle quantity	5
Guadalupe-Grau et al. (2017)	9 males (aged 84 ± 3 years) with low to severe COPD. Participants were overweight according to BMI, and 4/9 were sarcopenic.	Observational cohort study	No	9 weeks	18	Strength training plus HIIT. HIIT commenced from the third week: 5 min warm-up preceded 4 × 15 s, progressing to 5 × 25 s intervals with 60 s rest on a cycle ergometer.	“Sprints” at 80–90% HRR	>80% inclusion criteria. 14 started. 9 completed.	Not Not reported	Physical performance Muscle function	2
Hayes et al. (2017)	22 sedentary but otherwise healthy, males (62 ± 2 years)	Observational cohort study with MICT phase	No	6 weeks HIIT preceded by 6 weeks MICT	9 HIIT sessions	6 × 30 s intervals with 3 min rest on a cycle ergometer.	40% PPO or ~141% power achieved during a ramped CPET protocol to failure.	100% adherence	Not reported	Muscle quantity	2
Herbert et al. (2017a)	22 sedentary but otherwise healthy, males (62 ± 2 years) 17 male masters athletes (60 ± 5 years)	Observational cohort study with MICT phase	No	6 weeks HIIT preceded by 6 weeks MICT	9 HIIT sessions	6 × 30 s intervals with 3 min rest on a cycle ergometer.	40% PPO or ~141% power achieved during a ramped CPET protocol to failure.	100% adherence	Not reported	Muscle quantity	2
Herbert et al. (2017b)	17 male masters athletes (60 ± 5 years)	Observational cohort study	No	6 weeks HIIT preceded by 6 weeks MICT	9 HIIT sessions	6 × 30 s intervals with 3 min rest on a cycle ergometer.	40% PPO or ~141% power achieved during a ramped CPET protocol to failure.	100% adherence	Not reported	Muscle function	2
Hurst et al. (2019c)	36 untrained older adults, who were disease free (*n* = 18 HIIT; aged ~62 years, *n* = 18 control; aged ~63 years).	RCT	The study was registered with ClinicalTrials.gov	12 weeks	24	6 min warm-up preceded 4 sets of 4 resistance exercises. The programme was progressed	>90% peak heart rate was targeted. 89% peak heart rate achieved. Mean heart rate was 82% maximum.	>90% inclusion criteria. 99% achieved.	None attributed to the intervention.	Muscle function	7
Hwang et al. (2016)	51 untrained older adults, who were disease free (*n* = 15 completed HIIT; aged 65 ± 1 years, *n* = 15 completed control; aged 64 ± 2 years).	RCT	No	8 weeks	32	10 min warm-up preceded 4 × 4 min intervals with 3 min rest of synchronous arm and leg exercise on a non-weight bearing all-extremity air-braked ergometer. The programme was progressed.	>90% peak heart rate.	84% completed the study. Of those who completed the study, 89% attendance was achieved for HIIT.	None attributed to the intervention.	Muscle quantity	6
Jiménez-García et al. (2019)	82 healthy older adults 68 ± 5 years of age (*n* = 26 in HIIT)	RCT	The study was registered with ClinicalTrials.gov	12 weeks	24	10 min warm-up preceded 4 × 4 min suspension squats with 3 min rest.	90–95% peak heart rate.	>80% attendance as inclusion criteria.	None attributed to the intervention.	Physical performance	8
Losa-Reyna et al. (2019)	20 pre-frail or frail patients without multiple comorbidities, 84 ± 5 years of age (*n* = 11 in HIIT)	Quasi-experimental, non-randomized, single-blinded controlled study	No	6 weeks	12	Resistance training plus HIIT. 5 min warm-up preceded resistance exercise, and then 6–10 × 10–30 s with 40–100 s rest on a treadmill. The programme was progressed	90% maximal gait speed	16 started, 11 finished.	Not reported	Physical performance Muscle function	5
Malin et al. (2018)	Sedentary obese subjects (61 ± 3 years)	RCT with MICT as control	No	2 weeks	12	10 × 3 min intervals with 4 min rest on a cycle ergometer. The programme was progressed	90% peak heart rate	Not reported	Not reported	Muscle quantity	5
Martins et al. (2018)	16 postmenopausal sedentary women at high risk of type II diabetes (*n* = 8 HIIT; aged 64 ± 7 years, *n* = 8 combined training; aged 65 ± 6 years).	RCT with combined training (resistance and aerobic) as control	The study was registered with ClinicalTrials.gov	12 weeks	36	5 min warm-up preceded 10 × 60 s with 60 s rest bodyweight squats and steps. The programme was progressed	>85% peak heart rate	14 started, 8 finished.	Not reported	Muscle quantity Physical performance Muscle function	5
Nunes et al. (2019)	24 postmenopausal obese sedentary women (*n* = 12 HIIT; aged ~63 years, *n* = 12 combined training; aged ~63 years).	RCT with combined training (resistance and aerobic) as control	The study was registered with ClinicalTrials.gov	12 weeks	36	5 min warm-up preceded 10 × 60 s with 60 s rest bodyweight squats and steps. The programme was progressed	>85% peak heart rate	13 started, 12 finished. 91% adherence	Not reported	Muscle quantity Physical performance Muscle function	5
Robinson et al. (2017)	8 untrained older adults (71 ± 6 years), disease free, non-smokers.	Observational cohort study with sedentary control phase, followed by randomization into HIIT, combined training (resistance and aerobic), or resistance only training.	The study was registered with ClinicalTrials.gov	12 weeks	36	4 × 4 min with 3 min rest on a cycle ergometer.	>90% VO_2peak_	27 started, 23 finished.	Not reported	Muscle quantity Muscle function	3
Sculthorpe et al. (2017)	22 sedentary older males (62 ± 4 years), disease free.	RCT	No	12 weeks, of which 6 weeks was HIIT	9	5 min warm-up preceded 6 × 60 s with 3 min rest on a cycle ergometer.	40% PPO for the first 3 sessions, then 50% PPO for the remaining 6 sessions.	100% adherence.	None attributed to the intervention.	Muscle quantity Muscle function	5
Snijders et al. (2019)	14 sedentary men (74 ± 8 years), disease free, non-smokers.	Observational cohort study	The study was registered with ClinicalTrials.gov	12 weeks	36	Resistance training plus HIIT 3 min warm-up preceded 10 x 60 s with 60 s rest on a cycle ergometer.	~90% peak heart rate	Not reported	Not reported	Muscle function Muscle quantity	2
Søgaard et al. (2018)	22 sedentary older adults (aged 63 ± 1 years), disease free, non-smokers.	Observational cohort study	No	6 weeks	18	2 min warm-up preceded 5 × 60 s with 90 s rest on a cycle ergometer.	>85% power achieved during a ramped protocol to failure (individualized so participants could maintain intensity for 60 s).	28 started, 22 finished.	Not reported	Muscle quantity	2
Søgaard et al. (2019)	22 sedentary older adults (aged 63 ± 1 years), disease free, non-smokers.	Observational cohort study	No	6 weeks	18	2 min warm-up preceded 5 × 60 s with 90 s rest on a cycle ergometer.	>85% power achieved during a ramped protocol to failure (individualized so participants could maintain intensity for 60 s).	Not reported	Not reported	Muscle quantity	2
Taylor et al. (2019)	29 older adults (aged 64 ± 8 years) split into HIIT and MICT.	RCT with MICT as control	No	12 weeks	36	Not reported	Not reported	Not reported	Not reported	Muscle quantity	4
Wyckelsma et al. (2017)	15 older adults (aged 69 ± 4 years) disease free.	Observational cohort study	No	12 weeks	36	3 min warm-up preceded 4 × 4 min with 4 min rest on a cycle ergometer.	90–95% peak heart rate.	Not reported	Not reported	Muscle quantity	2

*RCT, randomized control trial; MICT, moderate intensity continuous training; SIT, sprint interval training; HIIT, high intensity interval training*.

### HIIT and Muscle Function

There were 20 studies which examined the effect of HIIT on muscle function using one or more of the criteria outline by EWGSOP (Cruz-Jentoft et al., 2019) (Table 2). Of these, 18 measured muscle strength, and five measured muscle power (some studies measured both, thus why this total is not 20). Of those reporting strength, six used the handgrip test, one used a 30 s arm curl test, five used a 30 s chair stand test, four used the 5 repetitions chair stand test, one used a 10 repetition chair stand test, two used knee extensor isokinetic dynamometry, one used a strain gauge for the knee extensors, four used a leg press, two used a chest press, three used a knee extension machine (which was not a dynamometer), and one used *latissimus dorsi* pull-down, horizontal row, and shoulder press. Of the 20 studies examining strength outcomes, 15 reported ≥1 strength parameter having been improved by HIIT compared to pre-training or compared to a moderate intensity continuous training (MICT) or non-exercise control. Of the remaining three (Robinson et al., 2017; Martins et al., 2018; Nunes et al., 2019), they all reported strength had improved more in a combined aerobic and resistance training group than a HIIT group.

**Table 2:** Summary of study details concerning HIIT and muscle function.

**Reference**	**Method of outcome measurement**	**Summary of results**
**MUSCLE STRENGTH**		
Aboarrage Junior et al. (2018)	30 s chair stand test	➚ **vs. pre-HIIT**, ➚ **vs. control** HIIT group 30 s chair stand test was 16 ± 4 repetitions and 19 ± 5 repetitions pre- and post-intervention, respectively. Control group 30 s chair stand test was 20 ± 2 repetitions and 19 ± 2 repetitions pre- and post-intervention, respectively.
Adamson et al. (2014)	5 rep chair stand test	➚ **vs. pre-HIIT**, ➚ **vs. control** HIIT group 5 rep chair stand test was 10.5 ± 2.2 s and 9.0 ± 1.6 s pre- and post-intervention, respectively. Control group 5 rep chair stand test was 12.1 ± 4.9 s and 11.9 ± 4.0 s pre- and post-intervention, respectively.
Adamson et al. (2020)	5 rep chair stand test	➚ **vs. pre-HIIT**, ➚ **vs. control** HIIT once weekly group 5 rep chair stand test was 11.9 ± 1.8 and 10.6 ± 2.1 s pre- and post-intervention, respectively. HIIT twice weekly group 5 rep chair stand test was 12.0 ± 2.1 s and 9.3 ± 1.1 s pre- and post-intervention, respectively. Control group 5 rep chair stand test was 12.1 ± 4.3 and 12.3 ± 4.2 s pre- and post-intervention, respectively.
Ballesta-García et al. (2019)	30 s arm curl test 30 s chair stand test	➚ **vs. pre-HIIT**, ➚ **vs. control**, ➚ **vs. MICT** HIIT group 30 s arm curl test was 28.9 ± 5.2 repetitions and 31.7 ± 5.5 repetitions pre- and post-intervention, respectively. Control group 30 s arm curl test was 20.6 ± 3.0 repetitions and 22.4 ± 2.9 repetitions pre- and post-intervention, respectively. MICT group 30 s arm curl test was 25.6 ± 5.2 repetitions and 25.1 ± 4.1 repetitions pre- and post-intervention, respectively. HIIT group 30 s chair stand test was 15.1 ± 2.7 repetitions and 20.7 ± 3.2 repetitions pre- and post-intervention, respectively. Control group 30 s chair stand test was 16.8 ± 2.9 repetitions and 14.9 ± 2.9 repetitions pre- and post-intervention, respectively. MICT group 30 s chair stand test was 13.7 ± 3.4 repetitions and 17.5 ± 4.9 repetitions pre- and post-intervention, respectively.
Bartlett et al. (2018)	30 s chair stand test Handgrip strength	➚ **vs. pre-HIIT** 30 s chair stand test was 14 ± 4 repetitions and 17 ± 5 repetitions pre- and post-HIIT, respectively. ➞ **vs. pre-HIIT** Handgrip strength was 18.3 ± 7.2 and 19.0 ± 8.1 kg pre- and post-HIIT, respectively.
Bruseghini et al. (2015)	Knee extensor isokinetic dynamometry.	➚ **vs. pre-HIIT**, ➘ **vs. resistance training** HIIT group isometric knee extensor torque at 60° knee flexion was 200 ± 21 Nm and 215 ± 32 Nm pre- and post-intervention, respectively. Resistance training group isometric knee extensor torque at 60° knee flexion was 202 ± 23 Nm and 223 ± 39 Nm pre- and post-intervention, respectively. ➞ **vs. pre-HIIT**, ➘ **vs. resistance training** HIIT group isometric knee extensor torque at 90° knee flexion was 169 ± 34 and 165 ± 31 Nm pre- and post-intervention, respectively. Resistance training group isometric knee extensor torque at 90° knee flexion was 166 ± 38 and 177 ± 42 Nm pre- and post-intervention, respectively. HIIT group concentric knee extensor torque at 60°·s^−1^ was 160 ± 24 and 163 ± 22 pre- and post-intervention, respectively. Resistance training group concentric knee extensor torque at 60°·s^−1^ was 164 ± 26 and 179 ± 31 Nm pre- and post-intervention, respectively. HIIT group concentric knee extensor torque at 120°·s^−1^ was 130 ± 23 and 133 ± 24 pre- and post-intervention, respectively. Resistance training group concentric knee extensor torque at 120°·s^−1^ was 132 ± 23 and 139 ± 23 Nm pre- and post-intervention, respectively.
Bruseghini et al. (2019)	Knee extensor isokinetic dynamometry.	➞ **vs. pre-HIIT**, ➘ **vs. resistance training** Knee extensor isokinetic dynamometry results at 90° knee flexion and 120°·s^−1^ are identical to Bruseghini et al. (2015).
Buckinx et al. (2018)	10 rep chair stand test	➚ **vs. pre-HIIT** 10 rep chair stand test was 18.8 ± 3.7 and 15.6 ± 3.7 s pre- and post-HIIT, respectively.
Buckinx et al. (2019)	Handgrip strength Knee extensor isometric strength using a chain-mounted strain gauge.	➚ **vs. pre-HIIT** Relative handgrip strength was 0.41 ± 0.11 and 0.43 ± 0.12 kg·kg^−1^ pre- and post-HIIT respectively, in a low protein group. Relative handgrip strength was 0.40 ± 0.09 and 0.41 ± 0.08 kg·kg^−1^ pre- and post-HIIT respectively, in a high protein group. ➞ **vs. pre-HIIT** Relative knee extensor isometric strength was 9.8 ± 2.5 and 10.1 ± 1.9 N·kg^−1^ pre- and post-HIIT, respectively, in a low protein group. Relative knee extensor isometric strength was 10.2 ± 1.6 and 10.4 ± 1.6 N·kg^−1^ pre- and post-HIIT, respectively, in a high protein group.
Coswig et al. (2020)	30 s chair stand test	➚ **vs. pre-HIIT**, ➚ **vs. MICT** HIIT group 30 s chair stand test was 8.4 ± 1.4 repetitions and 11.8 ± 2.1 repetitions pre- and post-intervention, respectively. MICT group 30 s chair stand test was 8.5 ± 1.1 repetitions and 11.0 ± 1.6 repetitions pre- and post-intervention, respectively.
Guadalupe-Grau et al. (2017)	30 s chair stand test Upper- and lower-limb isometric strength using a hydraulic hand dynamometer. 3 RM leg press and chest press. Handgrip strength	➚ **vs. pre-HIIT** 30 s chair stand test was 11.9 ± 4.2 repetitions and 17.0 ± 3.8 repetitions pre- and post-HIIT, respectively. Shoulder abduction strength was 10.9 ± 3.8 and 15.8 ± 4.3 kg pre- and post-HIIT, respectively. Hip flexion strength was 14.8 ± 3.7 and 21.1 ± 4.7 kg pre- and post-HIIT, respectively. Leg extension strength was 11.9 ± 2.1 and 18.2 ± 2.8 kg pre- and post-HIIT, respectively. 1-RM leg press strength was ~90 ± 20 and 145 ± 10 kg pre- and post-HIIT, respectively. 1-RM chest press strength was ~22 ± 8 and 40 ± 10 kg pre- and post-HIIT, respectively. ➞ **vs. pre-HIIT** Handgrip strength was 28.4 ± 5.0 and 30.3 ± 5.2 kg pre- and post-HIIT, respectively.
Hurst et al. (2019c)	Handgrip strength	➞ **vs. pre-HIIT**, ➞ **vs. control**, HIIT group handgrip strength was 36.2 ± 10.9 and ~38.1 kg pre- and post-intervention, respectively. Control group handgrip strength was 33.9 ± 11.0 and ~ 33.4 kg pre- and post-intervention, respectively.
Jiménez-García et al. (2019)	Handgrip strength	➚ **vs. pre-HIIT**, ➚ **vs. control**, ➚ **vs. MICT** HIIT group handgrip strength was ~25 ± 1 and ~28 ± 2 kg pre- and post-intervention, respectively. Control group handgrip strength was ~27 ± 2 and ~27 ± 2 kg pre- and post-intervention, respectively. HIIT group handgrip strength was ~25 ± 2 and ~26 ± 2 kg pre- and post-intervention, respectively.
Losa-Reyna et al. (2019)	5 rep chair stand test Leg-press force-velocity testing and 1-RM Handgrip strength	➚ **vs. pre-HIIT**, ➚ **vs. control** HIIT group 5 rep chair stand test was 15.6 ± 2.7 and 10.8 ± 2.5 s pre- and post-intervention, respectively. Control group 5 rep chair stand test was 15.7 ± 3.0 and 14.8 ± 4.0 s pre- and post-intervention, respectively. HIIT group handgrip strength was 16.3 ± 3.6 and 18.3 ± 2.3 kg pre- and post-intervention, respectively. Control group handgrip strength was 20.8 ± 6.0 and 20.1 ± 5.7 kg pre- and post-intervention, respectively. ➚ **vs. pre-HIIT** 1-RM leg-press strength was 49.2 ± 19.0 and 62.4 ± 23.2 kg pre- and post-HIIT, respectively. Load at peak power leg-press was 36.3 ± 18.1 and 42.3 ± 17.4 kg pre- and post-HIIT, respectively.
Martins et al. (2018)	1-RM unilateral knee extension.	➚ **vs. pre-HIIT**, ➘ **vs. combined training** HIIT group unilateral knee extension strength was 56.2 ± 17.7 and 56.8 ± 21.9 kg pre- and post-intervention, respectively. Combined training group unilateral knee extension strength was 47.8 ± 8.5 and 64.0 ± 64.0 kg pre- and post-intervention, respectively.
Nunes et al. (2019)	5 rep chair stand test 1-RM unilateral knee extension.	➚ **vs. pre-HIIT**, ➞ **vs. combined training** HIIT group 5 rep chair stand test was 12.3 (10.2–14.5) s and 9.3 (7.5–11.1) s pre- and post-intervention, respectively. Combined training group 5 rep chair stand test was 11.0 (9.7–12.4) s and 7.8 (6.8–8.8) s pre- and post-intervention, respectively. ➚ **vs. pre-HIIT**, ➘ **vs. combined training** HIIT group unilateral knee extension strength was 57.9 (47.6–68.1) and 61.5 (45.7–77.2) kg pre- and post-intervention, respectively. Combined training group unilateral knee extension strength was 50.7 (41.1–60.3) and 65.4 (54.8–75.9) kg pre- and post-intervention, respectively.
Robinson et al. (2017)	1-RM leg press.	➚ **vs. pre-HIIT**, ➘ **vs. combined training**, ➘ **vs. resistance training** HIIT group increased 1-RM leg press ~1.0 kg·FFM${}_{\text{leg}}^{-1}$ from pre- to post-intervention. Combine training group increased 1-RM leg press ~3.5 kg·FFM${}_{\text{leg}}^{-1}$ from pre- to post-intervention. Resistance training group increased 1-RM leg press ~4.3 kg·FFM${}_{\text{leg}}^{-1}$ from pre- to post-intervention.
Snijders et al. (2019)	1-RM leg press, chest press, *latissimus dorsi* pull-down, horizontal row, shoulder press, and knee extension.	➚ **vs. pre-HIIT** 1-RM leg press was 72 ± 25 and 92 ± 35 kg pre- and post-HIIT, respectively. 1-RM chest press was 21 ± 6 and 24 ± 7 kg pre- and post-HIIT, respectively. 1-RM *latissimus dorsi* pull-down was 26 ± 4 and 30 ± 5 kg pre- and post-HIIT, respectively. 1-RM knee extension was 27 ± 8 and 35 ± 9 kg pre- and post-HIIT, respectively. ➞ **vs. pre-HIIT** 1-RM shoulder press was 24 ± 7 and 27 ± 8 kg pre- and post-HIIT, respectively. 1-RM horizontal row was 28 ± 9 and 29 ± 5 kg pre- and post-HIIT, respectively.
**MUSCLE POWER**		
Buckinx et al. (2018)	Leg extensor power.	➚ **vs. pre-HIIT** Leg extensor power was 155 ± 70 and 186 ± 69 W pre- and post-HIIT, respectively.
Herbert et al. (2017a)	Peak power output, determined by a 6 s sprint on a cycle ergometer.	➚ **vs. pre-HIIT** Peak power output was 766 ± 163 and 856 ± 211 W pre- and post-HIIT, respectively.
Hurst et al. (2019c)	Leg extensor power.	➚ **vs. pre-HIIT**, ➚ **vs. control**, HIIT group leg extensor power was 159 ± 65 W and ~165 W pre- and post-intervention, respectively. Control group leg extensor power was 162 ± 63 and ~162 W pre- and post-intervention, respectively.
Losa-Reyna et al. (2019)	Leg-press force-velocity testing. 5 rep chair stand test power	➚ **vs. pre-HIIT**, ➚ **vs. control** HIIT group 5 rep chair stand power was 104 ± 32 and 156 ± 50 W pre- and post-intervention, respectively. Control group 5 rep chair stand test was 123 ± 23 and 134 ± 35 W pre- and post-intervention, respectively. ➚ **vs. pre-HIIT** HIIT group 5 leg-press peak power was 113 ± 62 and 153 ± 96 W pre- and post-intervention, respectively.
Sculthorpe et al. (2017)	Peak power output, determined by a 6 s sprint on a cycle ergometer.	➚ **vs. pre-HIIT**, ➚ **vs. control** HIIT group peak power output was 699 ± 180 and 831 ± 171 W pre- and post-intervention, respectively. Control group peak power output was 655 ± 130 and 657 ± 133 W pre- and post-intervention, respectively.

*1-RM, One repetition maximum; MICT, Moderate intensity continuous training; MIIT, Moderate intensity interval training ➚, superior to; ➘, worse than; ➞, equal to (according to statistical interpretation of original authors). Data are presented as mean ± standard deviation or mean (95% confidence intervals)*.

There were five studies which examined the effect of HIIT on muscle power, with two studies examining peak power output during cycle ergometry, and the remaining three determined power during a resistance training exercise (leg extension or leg-press). One investigation examined power during a 5 repetition chair stand test (Losa-Reyna et al., 2019). Of these studies, all reported improved power output post-HIIT. There was no evident association between change in muscle function (either 5 rep chair stand, 30 s chair stand, or grip strength) and number of bouts completed (Supplementary Figures 1a–c).

### HIIT and Muscle Quantity or Quality

There were 22 studies which examined the effect of HIIT on muscle quantity or a surrogate (fat free mass, lean mass, thigh volume; Table 3). Of these, 13 measured whole body lean mass by dual-energy X-ray absorptiometry (DEXA), nine measured leg lean mass by DEXA (of these, all nine also reported whole body lean mass), one measured whole body lean mass by air plethysmography, one measured *M. vastus lateralis* muscle thickness by ultrasonography, two measured quadriceps muscle volume by magnetic resonance imaging (MRI), two measured quadriceps cross-sectional area (CSA) or anatomical CSA (ACSA) by MRI, one measured whole body lean mass by MRI, one measured thigh muscle area by peripheral quantitative computed tomography (pQCT), and six measured whole body lean mass by bioelectrical impedance analysis (BIA). Of the 22 studies examining muscle quantity or quality outcomes, 11 reported ≥1 muscle quantity parameter was improved by HIIT, 14 reported no difference in ≥1 measure from pre-intervention or vs. a no exercise control, two reported inferior adaptation following HIIT compared to a group undertaking resistance training in ≥1 measure, one study reported lean mass was lost post-HIIT to a similar extent as a non-exercise control, and one did not report post-intervention lean mass (some studies measured several outcomes, thus why this total is not 22). There was no evident relationship between change in muscle quantity (as measured by lean mass) and number of bouts completed (Supplementary Figure 1d).

**Table 3:** Summary of study details concerning HIIT and muscle quantity or quality.

**Reference**	**Method of outcome measurement**	**Summary of results**
Aboarrage Junior et al. (2018)	Whole body lean mass by DEXA.	➞ **vs. control** HIIT group lean mass was 40 ± 6 and 41 ± 6 kg pre- and post-intervention, respectively. Control group lean mass was 40 ± 9 and 41 ± 10 kg pre- and post-intervention, respectively.
Andonian et al. (2018)	Whole body lean mass by air displacement plethysmography.	➞ **vs. pre-HIIT** Rheumatoid arthritis group lean mass was 44.9 ± 8.9 and 44.7 ± 7.8 kg pre- and post-HIIT, respectively. Prediabetes group lean mass was 50.1 ± 12.2 and 50.1 ± 12.0 kg pre- and post-HIIT, respectively.
Beetham et al. (2019)	Whole body and lower limb lean mass by DEXA.	➞ **vs. pre-HIIT**, ➞ **vs. MICT** HIIT group low limb lean mass was 17.6 ± 6.6 and ~17.2 kg pre- and post-intervention, respectively. MICT group lower limb lean mass was 17.0 ± 2.8 and ~17.3 kg pre- and post-intervention, respectively.
Boereboom et al. (2016)	Whole body and leg lean mass by DEXA. *M. vastus lateralis* muscle thickness determined by ultrasonography.	➞ **vs. pre-HIIT** Lean mass was 45.2 ± 11.1 and 45.3 ± 11.1 kg pre- and post-HIIT, respectively. ➚ **vs. pre-HIIT** Leg lean mass was 4.1 ± 1.3 and 4.2 ± 1.2 kg pre- and post-HIIT, respectively. *m. vastus lateralis* thickness was 2.04 ± 0.27 and 2.17 ± 0.28 cm pre- and post-HIIT, respectively.
Bruseghini et al. (2015)	Whole body and lower limb lean mass by DEXA. CSA and volume of the quadriceps by MRI.	➞ **vs. pre-HIIT**, ➘ **vs. resistance training** HIIT group lean mass was 56.9 ± 6.2 and 57.7 ± 5.3 kg pre- and post-intervention, respectively. Resistance training group lean mass was 57.3 ± 5.9 and 57.6 ± 5.8 kg pre- and post-intervention, respectively. ➚ **vs. pre-HIIT**, ➞ **vs. resistance training** HIIT group total quadriceps CSA was 60.3 ± 10.6 and 62.9 ± 10.5 cm^2^ pre- and post-intervention, respectively. Resistance training group total quadriceps CSA was 59.5 ± 9.3 and 62.0 ± 9.3 cm^2^ pre- and post-intervention, respectively. HIIT group total quadriceps volume was 820 ± 198 and 865 ± 199 cm^3^ pre- and post-intervention, respectively. Resistance training group total quadriceps volume was 812 ± 184 and 852 ± 188 cm^3^ pre- and post-intervention, respectively.
Bruseghini et al. (2019)	Volume and ACSA of the quadriceps by MRI.	➚ **vs. pre-HIIT**, ➞ **vs. resistance training** Total quadriceps volume results are identical to Bruseghini et al. (2015). HIIT group total quadriceps ACSA increased 3.09 ± 1.38, 2.27 ± 252, and 2.65 ± 3.04 cm^2^ at 25, 50, and 75% femur length, respectively, compared to pre-intervention. Resistance training group total quadriceps ACSA increased 3.19 ± 1.24, 3.03 ± 3.04, and 3.40 ± 3.21 cm^2^ at 25, 50, and 75% femur length, respectively compared to pre-intervention. ➞ **vs. pre-HIIT**, ➘ **vs. resistance training** HIIT group PCSA at 50% femur length was unchanged post-intervention. Resistance training group PCSA at 50% femur length increased post-intervention.
Buckinx et al. (2019)	Whole body and leg lean mass by DEXA. Thigh muscle area by pQCT.	➞ **vs. pre-HIIT** Lean mass was 51.8 ± 7.3 and 53.0 ± 7.9 kg pre- and post-HIIT, respectively, in a low protein group. Lean mass was 43.1 ± 9.3 and 43.4 ± 9.5 kg pre- and post-HIIT, respectively, in a high protein group. Leg lean mass was 18.4 ± 3.0 and 18.8 ± 3.3 kg pre- and post-HIIT, respectively, in a low protein group. Leg lean mass was 15.4 ± 3.5 and 15.7 ± 3.5 kg pre- and post-HIIT, respectively, in a high protein group. Thigh muscle area was 91.8 ± 11.9 and 94.4 ± 15.6 cm^2^ pre- and post-HIIT, respectively, in a low protein group. Thigh muscle area was 99.3 ± 21.7 cm^2^ and 95.7 ± 21.8 cm^2^ pre- and post-HIIT, respectively, in a high protein group.
Coswig et al. (2020)	Whole body lean mass by BIA.	➞ **vs. pre-HIIT**, ➞ **vs. MICT** HIIT group lean mass was 29.4 ± 2.8 and 29.6 ± 2.7 kg pre- and post-intervention, respectively. MICT group lean mass was 30.1 ± 3.5 and 29.9 ± 3.6 kg pre- and post-intervention, respectively.
Hayes et al. (2017)	Whole body lean mass by BIA.	➚ **vs. pre-HIIT** Lean mass was 66.7 ± 7.1 and 69.1 ± 8.3 kg pre- and post-HIIT, respectively.
Herbert et al. (2017b)	Whole body lean mass by BIA.	➚ **vs. pre-HIIT** Sedentary group lean mass was 66.7 ± 7.1 and 69.1 ± 8.3 kg pre- and post-HIIT, respectively. Masters athlete group lean mass was 65.2 ± 6.4 and 67.9 ± 5.1 kg pre- and post-HIIT, respectively.
Hwang et al. (2016)	Whole body lean mass by DEXA.	➞ **vs. pre-HIIT**, ➞ **vs. control**, ➞ **vs. MICT** HIIT group lean mass was 44.6 ± 2.6 and 45.0 ± 2.4 kg pre- and post-intervention, respectively. MICT group lean mass was 47.8 ± 2.1 and 47.7 ± 1.9 kg pre- and post-intervention, respectively. Control group lean mass was 48.3 ± 2.9 and 48.4 ± 2.9 kg pre- and post-intervention, respectively.
Jiménez-García et al. (2019)	Whole body lean mass by BIA.	➞ **vs. pre-HIIT**, ➞ **vs. control**, ➞ **vs. MIIT** HIIT group lean mass was 24.9 ± 5.7 and 25.7 ± 6.7 kg pre- and post-intervention, respectively. MIIT group lean mass was 25.6 ± 6.6 and 24.5 ± 6.3 kg pre- and post-intervention, respectively. Control group lean mass was 24.6 ± 4.8 and 23.8 ± 4.5 kg pre- and post-intervention, respectively.
Malin et al. (2018)	Whole body lean mass by BIA.	➘ **vs. pre-HIIT**, ➞ **vs. control** HIIT group lean mass decreased 0.4 ± 0.1 kg from pre- to post-intervention. Control group lean mass decreased 0.4 ± 0.1 kg from pre- to post-intervention
Martins et al. (2018)	Whole body lean mass by DEXA, expressed as muscle mass index.	➚ **vs. pre-HIIT**, ➞ **vs. combined training** HIIT group muscle mass index was 6.6 ± 0.7 and 6.8 ± 0.9 kg·m^2^ pre- and post-intervention, respectively. Combined training group muscle mass index was 6.6 ± 1.1 kg and 6.8 ± 1.3 kg·m^2^ pre- and post-intervention, respectively.
Nunes et al. (2019)	Whole body and leg lean mass by DEXA.	➞ **vs. pre-HIIT**, ➞ **vs. combined training** HIIT group lean mass was 37.5 (33.9–41.1) kg and 37.5 (33.8–41.2) kg pre- and post-intervention, respectively. Combined training group lean mass was 36.0 (32.7–39.2) kg and 36.3 (32.8–39.8) kg pre- and post-intervention, respectively. ➚ **vs. pre-HIIT**, ➞ **vs. combined training** HIIT group leg lean mass was 12.7 (11.1–14.2) kg and 12.9 (11.3–14.6) kg pre- and post-intervention, respectively. Combined training group leg lean mass was 12.3 (10.8–13.8) kg and 12.7 (11.1–14.4) kg pre- and post-intervention, respectively.
Robinson et al. (2017)	Whole body lean mass by DEXA.	➚ **vs. pre-HIIT**, ➞ **vs. combined training**, ➞ **vs. resistance training** HIIT group increased fat free mass ~0.9 kg from pre- to post-intervention. Combine training group increased fat free mass ~1.0 kg from pre- to post-intervention. Resistance training group increased fat free mass ~1.2 kg from pre- to post-intervention.
Sculthorpe et al. (2017)	Whole body lean mass by BIA.	➚ **vs. pre-HIIT**, ➚ **vs. control** HIIT group lean mass was 65.9 ± 6.7 and 68.1 ± 7.5 kg pre- and post-intervention, respectively. Control group lean mass was 63.4 ± 6.9 and 63.6 ± 7.3 kg pre- and post-intervention, respectively.
Snijders et al. (2019)	Whole body and leg lean mass by DEXA.	➞ **vs. pre-HIIT** Lean mass was 55.0 ± 7.8 kg and 55.3 ± 7.7 kg pre- and post-HIIT, respectively. Leg lean mass was 19.3 ± 3.6 kg and 19.5 ± 3.4 kg pre- and post-HIIT, respectively.
Søgaard et al. (2018)	Whole body and leg lean mass by DEXA.	➞ **vs. pre-HIIT** Female lean mass was 43.3 ± 1.0 and 43.7 ± 1.0 kg pre- and post-HIIT, respectively. Male lean mass was 59.6 ± 2.0 and 60.0 ± 2.0 kg pre- and post-HIIT, respectively. Female leg lean mass was 15.5 ± 0.4 kg and 15.5 ± 0.5 kg pre- and post-HIIT, respectively. Male leg lean mass was 21.0 ± 0.7 and 21.2 ± 0.7 kg pre- and post-HIIT, respectively.
Søgaard et al. (2019)	Whole body and leg lean mass by DEXA.	➚ **vs. pre-HIIT** Lean mass was 51.5 ± 2.1 and 51.8 ± 2.1 kg pre- and post-HIIT, respectively.
Taylor et al. (2019)	Whole body lean mass by MRI.	➚ **vs. pre-HIIT**, ➞ **vs. MICT** HIIT group increased fat free mass 0.3 ± 0.9 kg from pre- to post-intervention. MICT group increased fat free mass 0.9 ± 1.5 kg from pre- to post-intervention.
Wyckelsma et al. (2017)	Whole body and leg lean mass by DEXA.	Data not reported post-intervention

*DEXA, Dual-energy X-ray absorptiometry; MRI, Magnetic resonance imaging; CSA, Cross sectional area; ACSA, Anatomical cross-sectional area; pQCT, peripheral quantitative computed tomography; BIA, bioelectrical impedance analysis; MICT, Moderate intensity continuous training; MIIT, Moderate intensity interval training; ➚, superior to; ➘, worse than; ➞, equal to (according to statistical interpretation of original authors). Data are presented as mean ± standard deviation or mean (95% confidence intervals)*.

### HIIT and Physical Performance

There were 12 studies which examined the effect of HIIT on physical function (Table 4). One used the short physical performance battery (SPPB), eight used gait speed or the 6 min walk test (6MWT), nine used the timed up and go (TUG) test, and one used the 400 m walk test (some studies utilized more than one outcome). Of the 12 studies examining physical performance, all reported ≥1 parameter was improved by HIIT. The only study examining SPPB reported HIIT improved SPPB performance.

**Table 4:** Summary of study details concerning HIIT and physical performance.

**Reference**	**Method of outcome measurement**	**Summary of results**
Aboarrage Junior et al. (2018)	TUG	➚ **vs. pre-HIIT**, ➚ **vs. control** HIIT group TUG was 6.86 ± 1.24 and 6.22 ± 1.13 s pre- and post-intervention, respectively. Control group TUG was 5 ± 1 and 6 ± 1 s pre- and post-intervention, respectively.
Adamson et al. (2014)	TUG	➚ **vs. pre-HIIT**, ➚ **vs. control** HIIT group TUG was 6.5 ± 0.8 and 5.8 ± 0.6 s pre- and post-intervention, respectively. Control group TUG was 6.9 ± 1.0 and 6.7 ± 1.0 s pre- and post-intervention, respectively.
Adamson et al. (2020)	TUG	➚ **vs. pre-HIIT**, ➚ **vs. control** HIIT once weekly group TUG was 6.7 ± 0.9 and 6.2 ± 0.7 s pre- and post-intervention, respectively. HIIT twice weekly TUG was 7.0 ± 1.2 and 5.9 ± 0.5 s pre- and post-intervention, respectively. Control group TUG was 7.0 ± 1.1 and 6.7 ± 1.1 s pre- and post-intervention, respectively.
Ballesta-García et al. (2019)	TUG 6MWT	➚ **vs. pre-HIIT**, ➚ **vs. control** HIIT group TUG was 6.08 ± 1.31 and 5.30 ± 0.80 s pre- and post-intervention, respectively. MICT group TUG was 6.40 ± 1.23 and 5.53 ± 1.28 s pre- and post-intervention, respectively. Control group TUG was 5.89 ± 0.74 and 6.25 ± 0.89 s pre- and post-intervention, respectively. HIIT group 6MWT was 564 ± 41.0 and 600 ± 74.9 m pre- and post-intervention, respectively. MICT group 6MWT was 502 ± 72.3 and 545 ± 72.6 m pre- and post-intervention, respectively. Control group 6MWT was 510 ± 59.0 and 494 ± 49.5 m pre- and post-intervention, respectively.
Bartlett et al. (2018)	TUG 400 m walk	➞ **vs. pre-HIIT** TUG was 8.8 ± 1.8 and 8.4 ± 1.9 s pre- and post-intervention, respectively. ➚ **vs. pre-HIIT** 400 m walk was 251 ± 62 and 233 ± 51 s pre- and post-intervention, respectively.
Buckinx et al. (2018)	TUG 6MWT	➚ **vs. pre-HIIT** HIIT group TUG was 7.5 ± 1.1 and 6.6 ± 0.9 s pre- and post-intervention, respectively. HIT group 6MWT was 550 ± 85 and 618 ± 91 m pre- and post-intervention, respectively.
Coetsee and Terblanche (2017)	TUG	➞ **vs. pre-HIIT**, ➞ **vs. control**, ➞ **vs. MICT**, ➞ **vs. resistance training** HIIT group TUG was 5.6 ± 0.7 and 5.3 ± 0.7 s pre- and post-intervention, respectively. Control group TUG was 5.5 ± 1.1 and 5.7 ± 0.8 s pre- and post-intervention, respectively. MICT group TUG was 5.6 ± 0.7 and 5.4 ± 0.8 s pre- and post-intervention, respectively. RT group TUG was 5.4 ± 0.9 and 5.1 ± 0.8 s pre- and post-intervention, respectively.
Coswig et al. (2020)	Gait speed (10 m) 6MWT	➞ **vs. pre-HIIT**, ➞ **vs. MIIT**, ➞ **vs. MICT** HIIT group gait velocity was 1.3 ± 0.1 and 1.3 ± 0.1 m·s^−1^ pre- and post-intervention, respectively. MIIT group gait velocity was 1.3 ± 0.1 and 1.2 ± 0.1 m·s^−1^ pre- and post-intervention, respectively. MICT group gait velocity was 1.3 ± 0.1 and 1.3 ± 0.1 m·s^−1^ pre- and post-intervention, respectively. ➚ **vs. pre-HIIT**, ➞ **vs. MIIT**, ➞ **vs. MICT** HIIT group 6MWT was 406 ± 74 and 454 ± 72 m pre- and post-intervention, respectively. MIIT group 6MWT was 403 ± 83 and 451 ± 84 m pre- and post-intervention, respectively. MICT group 6MWT was 413 ± 58 and 427 ± 68 m pre- and post-intervention, respectively.
Guadalupe-Grau et al. (2017)	TUG 6MWT	➚ **vs. pre-HIIT** TUG was 9.1 ± 1.6 and 7.0 ± 0.9 s pre- and post-intervention, respectively. 6MWT was 286.1 ± 107.2 and 396.2 ± 106.5 m pre- and post-intervention, respectively.
Jiménez-García et al. (2019)	Gait speed (via TUG test)	➚ **vs. pre-HIIT**, ➚ **vs. MIIT**, ➚ **vs. control** HIIT group gait speed was 0.73 and 0.89 m·s^−1^ pre- and post-intervention, respectively. MIIT group gait speed was 0.75 and 0.75 m·s^−1^ pre- and post-intervention, respectively. Control group gait sped was 0.75 and 0.75 m·s^−1^ pre- and post-intervention, respectively.
Losa-Reyna et al. (2019)	SPPB 6MWT	➚ **vs. pre-HIIT**, ➚ **vs. control** HIIT group SPPB was 6.8 ± 1.5 points and 9.8 ± 1.5 points pre- and post-intervention, respectively. Control group SPPB was 7.4 ± 2.0 points and 6.9 ± 2.7 points pre- and post-intervention, respectively. ➞ **vs. pre-HIIT** 6MWT was 257 ± 62 and 302 ± 72 m pre- and post-intervention, respectively. 6MWT was not performed in the control group.
Martins et al. (2018)	6MWT	➚ **vs. pre-HIIT**, ➞ **vs. combined training** HIIT group 6MWT was 577 ± 83 and 600 ± 92 m pre- and post-intervention, respectively. Combined training group 6MWT was 614 ± 89 and 669 ± 105 m pre- and post-intervention, respectively.

*TUG, timed up and go; 6MWT, 6-min walk test; SPPB, short physical performance battery; HIIT, high intensity interval training; MICT, moderate intensity continuous training; MIIT, Moderate intensity interval training; ➚, superior to; ➘, worse than; ➞, equal to (according to statistical interpretation of original authors). Data are presented as mean ± standard deviation or mean (95% confidence intervals)*.

There was no evident relationship between change in muscle performance (as measured by TUG and 6MWT) and number of bouts completed (Supplementary Figures 1e,f).

## Discussion

This scoping review provided an overview of existing literature pertaining to HIIT and phenotypic characteristics of sarcopenia. We examined outcomes according to the revised EWGSOP definition (Cruz-Jentoft et al., 2019) to facilitate translation of research findings into clinical practice. Firstly, the earliest article cited was Adamson et al. (2014) published in 2014, which speaks to this rapidly emerging area of research. This review catalogs existing literature, with a view to facilitating discussion of research opportunities and issues that need to be addressed in future studies.

In relation to our first objective, which was to search the literature for the effect of HIIT on phenotypic characteristics of sarcopenia in older adults, we observed most studies reported at least one positive change in characteristics when compared to vs. pre-HIIT, vs. non-exercise control, or vs. MICT. In this context, 19 of 20 studies reported an improvement to ≥1 muscle function outcome for ≥1 comparisons examined (vs. pre-HIIT, vs. non-exercise control, or vs. MICT) (Bruseghini et al., 2019). Similarly, twelve of 22 reported an improvement to ≥1 muscle quantity outcome for ≥1 comparison examined, and 11 of 12 reported an improvement to for ≥1 physical performance outcome for ≥1 comparison examined.

In relation to our second objective, training programmes ranged in duration from 2 to 24 weeks (median = 9.5 weeks), incorporated resistance training based HIIT, running/walking HIIT, cycling HIIT, and HIIT combined with other exercise modes (i.e., resistance training). Populations studied were commonly in the 7th decade of life, and mostly living independently. In relation to our third objective, muscle quantity, or quality was most frequently studied in the included literature. DEXA was the most utilized measurement method, which is in line with the EWGSOP algorithm for sarcopenia case findings in clinical practice (Cruz-Jentoft et al., 2019). However, these are only routinely found in research facilities and hospitals and would likely require a referral from primary care before an individual received a DEXA scan. Importantly, none of the included studies involved participants who had been diagnosed with sarcopenia using a formalized definition. This limits the clinical significance of the included literature and clearly highlights a need for further work in this population.

### HIIT and Muscle Function

According to the revised EWGSOP definition of sarcopenia (Cruz-Jentoft et al., 2019), muscle function is primarily considered as muscle strength. Yet, the chair stand test (or its variations) is named as a parameter that measures muscle strength. However, as the chair stand test relies on the ability to generate force over a short period of time, this could be considered a test of muscle power, rather than a measure of maximal force. The term *dynapenia* [i.e., the age-associated reduction in muscle strength and power (Clark and Manini, 2012; Manini and Clark, 2012)] was originally used to differentiate itself from sarcopenia (Clark and Manini, 2008), which has its roots in age-related reduced muscle mass [Greek translation = “poverty of flesh” (Kim and Choi, 2013)]. However, more recent definitions and diagnoses of sarcopenia have broadened to include muscle function. In this context, when one measures muscle strength using non-isometric movements (i.e., when work occurs), force, distance, and time can be extracted, which is quantification of power. Thus, we believed it pertinent to include studies which concerned muscle power within this review. In fact, muscle power associates more strongly with physical performance and independence than muscle quantity (Clark and Manini, 2010; Trombetti et al., 2016), which may explain why the chair stand test is at the forefront of the revised EWGSOP algorithm for diagnosing and quantifying sarcopenia (Cruz-Jentoft et al., 2019). Moreover, as this is a scoping review, our *a priori* aim was to outline the range and characteristics of outcome variables examined.

In this review, only six studies used grip strength as an outcome measure (Guadalupe-Grau et al., 2017; Buckinx et al., 2019; Hurst et al., 2019c; Jiménez-García et al., 2019). This is interesting to note as EWGSOP propose grip strength as the primary measurement of muscle strength in clinical practice and research studies (Cruz-Jentoft et al., 2019). However, of these six investigations, two were published before the revised EWGSOP guidelines, and four were published the same year, so data collection may have been pre-update. Wiśniowska-Szurlej et al. (2019) examined handgrip strength and other mobility parameters including gait speed, balance, and chair stand and observed weak correlations between handgrip strength and mobility in older adults under long-term care facilities. Yee et al. (2021) corroborated this finding reporting weak correlations between chair stand test and handgrip strength in community-dwelling older adults. Similarly, changes in handgrip strength do correlate with changes in leg muscle strength of physical performance during an exercise intervention program in frail older people (Tieland et al., 2015), suggesting it is not a good surrogate of mobility, muscle function, or change in muscle function of muscle other than those involved in gripping. If the two proposed measures of muscle strength to diagnose sarcopenia are not in agreement, then an alternative method for measuring muscle strength is necessary in this population. This may explain why most studies in this review have not measured handgrip and instead opted for isokinetic dynamometry, considered the gold standard for assessing muscle strength but not commonly used in a clinical setting. When considering the body of studies examining muscle function, the majority report increased strength (70% of studies) or power (100% of studies) following HIIT.

Considering reduced muscle function is at the forefront of the recent update on the definition and treatment of sarcopenia (Cruz-Jentoft et al., 2019), any intervention targeting the prevention or reversal of phenotypic characteristics of sarcopenia must be capable of enhancing muscle strength. To our knowledge, Losa-Reyna et al. (2019) is the only investigation to examine an exercise intervention containing HIIT in frail older adults. These authors examined the influence of a 6-week multicomponent exercise intervention (including walking-based HIIT) focused on enhancing muscle power in ~84-year olds (range 77–96 years; 75% females; 35% pre-frail and 65% frail). Post-intervention, leg press strength had improved by 34%, and muscle power improved by 47%. Moreover, load at peak power on the force-velocity curve increased by 23%, which suggests this type of intervention may improve muscle strength and power in frail and pre-frail elderly.

### HIIT and Muscle Quantity or Quality

In this review, 20/21 (95%) of studies report appendicular skeletal muscle mass measured by DEXA, BIA, or MRI, or cross-sectional area of the thigh by MRI or pQCT scan, which are the primary measurement of muscle quantity proposed by EWGSOP in clinical practice and research (Cruz-Jentoft et al., 2019). The remaining investigation used air plethysmography to determine whole body lean mass (Andonian et al., 2018). When considering the body of studies examining total body lean mass, several reported no increase from pre-HIIT (Bruseghini et al., 2015; Boereboom et al., 2016; Hwang et al., 2016; Andonian et al., 2018; Malin et al., 2018; Søgaard et al., 2018; Beetham et al., 2019; Buckinx et al., 2019; Jiménez-García et al., 2019; Nunes et al., 2019; Snijders et al., 2019; Coswig et al., 2020), whereas some reported an increase post-HIIT compared to pre-HIIT (Hayes et al., 2017; Herbert et al., 2017a; Sculthorpe et al., 2017). To add further uncertainty, two studies which observed no increase in whole body lean quantity observed increased thigh lean mass (Boereboom et al., 2016; Bruseghini et al., 2019). Taken together, it is unclear whether HIIT can significantly increase muscle quantity or quality, and the result may be determined by measurement technique of muscle quantity.

There are no data concerning the effect of HIIT on skeletal muscle quantity or its surrogates (e.g., fat free mass [FFM], lean body mass) in adults diagnosed with sarcopenia, or oldest old humans, despite emerging evidence in the rodent model (Seldeen et al., 2018). Thus, data from the middle old and young old must be extrapolated until these studies exist. In this context, and despite no changes in muscle strength, Robinson et al. (Robinson et al., 2017) observed a ~1 kg increase in FFM in sedentary ~71 year olds following 3 days/week cycling HIIT and 2 days/week of treadmill walking. This increase was greater in a resistance training only group, however. Interestingly, FFM was also increased to the same extend in a young (~25 years old) sedentary cohort, suggesting HIIT can increase FFM in the young and old to equal magnitude. This can be interpreted in two ways: 1) sedentary older adults maintain muscle plasticity and sensitivity to HIIT into older age, and 2) HIIT can increase FFM quantity in young sedentary adults who have not experienced muscle wastage. However, as all participants were untrained, increased FFM could be attributed to both young and old participants being HIIT-naïve.

It would have been a reasonable *a priori* hypothesis to predict HIIT performed at the greatest relative intensity (i.e., all-out or SIT) would result in the greatest increases in muscle quantity, as intensities closer to maximal voluntary contraction are known to induce muscle hypertrophy (Schoenfeld, 2010; Krzysztofik et al., 2019). However, this was not observed as Aboarrage Junior et al. (2018) utilized an all-out protocol, with no reported increases in lean mass. Likewise, it may have been expected untrained participants would exhibit the greatest increase in muscle quantity. However, Herbert et al. (2017a) examined the body composition changes in a group of previously sedentary older males and masters athletes, and reported FFM increased ~3% (from ~67 to ~69 kg) and ~4% (from ~65 to ~68 kg), respectively. This suggests HIIT may be efficacious at increasing FFM in highly active older males and previously sedentary older male, if they are HIIT-naïve. Yet, these data are not ubiquitous through the included literature of this review. Adequate intake of dietary protein is also an important consideration for older adults and any potential exercise induced increases in muscle mass are likely to be influenced by this (Beaudart et al., 2019).

### HIIT and Physical Performance

In this review, all of the studies assessing physical performance reported gait speed (part of the SPPB), the SPPB, or the TUG test as an outcome, which are the primary measurements of physical performance proposed by EWGSOP in clinical practice and research (Cruz-Jentoft et al., 2019). Four investigations also reported the 5 repetitions chair stand test separately (Adamson et al., 2014, 2020; Losa-Reyna et al., 2019; Nunes et al., 2019). However, this is one element of the SPPB, so those reporting SPPB values will have conducted this test. When considering the body of literature examining physical performance, all studies reported improvements post-HIIT. When considering studies examining physical performance, all studies report increased physical performance of ≥1 parameter following HIIT. In some instances HIIT did not improve performance more than another training method, where investigations had a parallel arm (Martins et al., 2018; Ballesta-García et al., 2019; Nunes et al., 2019). Physical performance represents a multidimensional construct involving a range of physiological systems across the whole-body (Beaudart et al., 2019) and is a key component in the definition of severe sarcopenia (Cruz-Jentoft et al., 2019).

Losa-Reyna et al. (2019) observed that a 6-week multicomponent exercise intervention (including walking-based HIIT) focused on enhancing muscle power improved the frailty phenotype by 1.6 points, muscle strength by 34%, and muscle power by 47%, suggesting this type of intervention is feasible in frail and pre-frail elderly. As this intervention was multicomponent, it is not possible to quantify the contribution of HIIT to the overall improvement, and therefore it is difficult to ascertain whether adaptations would have occurred were HIIT examined in isolation, rather than simultaneously with a resistance training programme.

### Strengths and Limitations

In cataloging the research concerning HIIT and phenotypic characteristics of sarcopenia, several issues and considerations came to light, all of which have important implications for the interpretation of this body of literature, and improvement of future investigations. Firstly, the use of exercise terminology requires clarity. In this context, we mean the definition of “HIIT.” HIIT has previously been described as periods of work >85% VO_2peak_ or 85% HR_max_ or equivalent perception-based approaches, interspersed by recovery periods (Gibala et al., 2012). Only articles matching this description were included in this article. Several articles were returned from our database searching which termed the exercise intervention HIIT, but often these did not reach this threshold of intensity. Similarly, when exercise is described as “all-out,” this should be termed SIT, which although a subcategory of HIIT, is unique in its prescription (Weston et al., 2014). It is imperative to classify protocols based on the nature of exercise prescription as different interval exercise classifications will alter experience and potentially subsequent adaptation to the exercise (Biddle and Batterham, 2015). Penultimately, the majority of studies considered small samples sized, which limits interpretation. Finally, the major limitation of the present scoping review is the lack of studies in older adults diagnosed with sarcopenia. Whilst the literature assessment was comprehensive, it is possible that studies may have been missed from the analysis, but as three databases were searched, it is unlikely enough were missed to create a large void in the included literature.

One questions that cannot be answered in the current scoping review is the effect of age on adaptations in physical performance, muscle function, or muscle quantity with HIIT. Whilst we attempted to examine results by decade (60–69, 70–79, and ≥80 years of age), it was noted that most published results were performed in “younger old” participants between 60 and 70 years of age. Further meta-analytical subgroup analysis or meta-regression may thus be required to examine differing responses by age group. In a similar manner, another limitation noted is the inability to examine potential sex differences in responses to HIIT for any outcome. Whilst most studies utilized both male and female participants, groups were typically mixed and thus no insight into sex difference of HIIT responses is attempted here. With a need to better describe and report female physiology in exercise physiology literature (Elliott-Sale et al., 2021), more work in this area may this be called for.

It is also important to acknowledge that the studies included in this review were delivered across a range of settings and involved a diverse range of older adults of varying health and fitness status. While this makes generalizing findings difficult, it does suggest that HIIT may be feasible across a broad range of settings with a wide range of older people. However, it is important to make clear that HIIT may not be suitable for all older people and all exercise programmes should be individually prescribed based on the characteristics of the individual.

### Recommendations for Advancement of the Investigative Area

In relation to our fourth objective (provide recommendations for the advancement of the investigative area), this review revealed a dearth of studies considering participants diagnosed with sarcopenia. Therefore, our primary recommendation for advancement of the research area is to increase studies that recruit participants or patients with sarcopenia, or those who are at risk from sarcopenia (i.e., the oldest old). These studies could be feasibility trials, as there is little information as to whether HIIT is a feasible exercise approach in older people. Secondly, given the issue regarding terminology and exercise intensity discussed above, authors are encouraged to be consistent in the use of exercise terminology by adhering to the consensus on exercise reporting template [CERT; (Slade et al., 2016)] in future investigations, which would permit assessment of intervention heterogeneity. Thirdly, studies included within this review had a sample size ranging from 8 to 82 participants, possibly due to resource commitments associated with having large sample sizes and/or rigorous research design. We suggest multicentre RCTs to improve (a) statistical power, and (b) the quality of available evidence, as only 17/32 studies achieved ≥5 on the PEDro scale. Finally, although this review focused directly on phenotypic characteristics of sarcopenia (i.e., quantitative assessment), qualitative investigations on the perceptions of adults with phenotypic characteristics of sarcopenia on this type of exercise and how it could be delivered to this population with minimizing any barriers will be beneficial for the field of gerontology.

## Conclusions and Practical Recommendations

In conclusion, most studies presented herein utilized outcome measures defined by the revised EWGSOP guidelines. There was divergence observed in exercise interventions, with HIIT interventions involving a range of exercise modes delivered in a range of settings. Currently, there is some evidence suggesting HIIT may improve phenotypic characteristics of sarcopenia. However, there are few studies investigating any form of HIIT in the very old, or those diagnosed with sarcopenia. Therefore, more intervention studies are needed in this population to confirm this phenomenon and confidently quantify the effectiveness of HIIT. In addition, we need to understand if this is a safe and feasible training approach in this population. In a practical context, combined interventions involving HIIT and resistance training are a worthy avenue for investigation as resistance training is the most potent stimulus to increase muscle quantity and studies herein showed divergent results concerning HIIT and muscle quantity. Finally, HIIT or SIT that is easy to apply (i.e., without equipment needs, travel, specialist training, and intensity monitoring such as heart rate or power output) or can be supported virtually is likely needed to promote the transition of HIIT from the laboratory to the real world.

## Data Availability Statement

The original contributions presented in the study are included in the article/Supplementary Material, further inquiries can be directed to the corresponding authors.

## Author Contributions

LH and CH: conceptualization, methodology, investigation, and project administration. LH and NS-H: formal analysis and investigation. LH, BE, ZY, TB, NS, NS-H, and CH: writing—original draft preparation. LH, BE, TB, NS, NS-H, and CH: writing—review and editing. LH and BE: visualization. LH and CH: funding acquisition. All authors contributed to the article and approved the submitted version.

## Conflict of Interest

The authors declare that the research was conducted in the absence of any commercial or financial relationships that could be construed as a potential conflict of interest.

## Publisher's Note

All claims expressed in this article are solely those of the authors and do not necessarily represent those of their affiliated organizations, or those of the publisher, the editors and the reviewers. Any product that may be evaluated in this article, or claim that may be made by its manufacturer, is not guaranteed or endorsed by the publisher.

## Supplementary Material

Supplementary Figure 1 Percent change (Δ%) in outcome measures for muscle function **(a)** 5 s chair stand, **(b)** 30 s chair stand, **(c)** grip strength, muss quality **(d)** lean mass, or muscle performance **(e)** timed up and go (TUG), and **(f)** 6 min walk test (6MWT), all as a function of the number of bouts completed. Dashed lines indicate 95% confidence intervals.
