# Supplementary figures and images for: High Intensity Interval Training (HIIT) as a Potential Countermeasure for Phenotypic Characteristics of Sarcopenia: A Scoping Review
Source: Front Physiol. 2021 Aug 24;12:715044. doi: 10.3389/fphys.2021.715044 (PMC8423251; doi:10.3389/fphys.2021.715044)

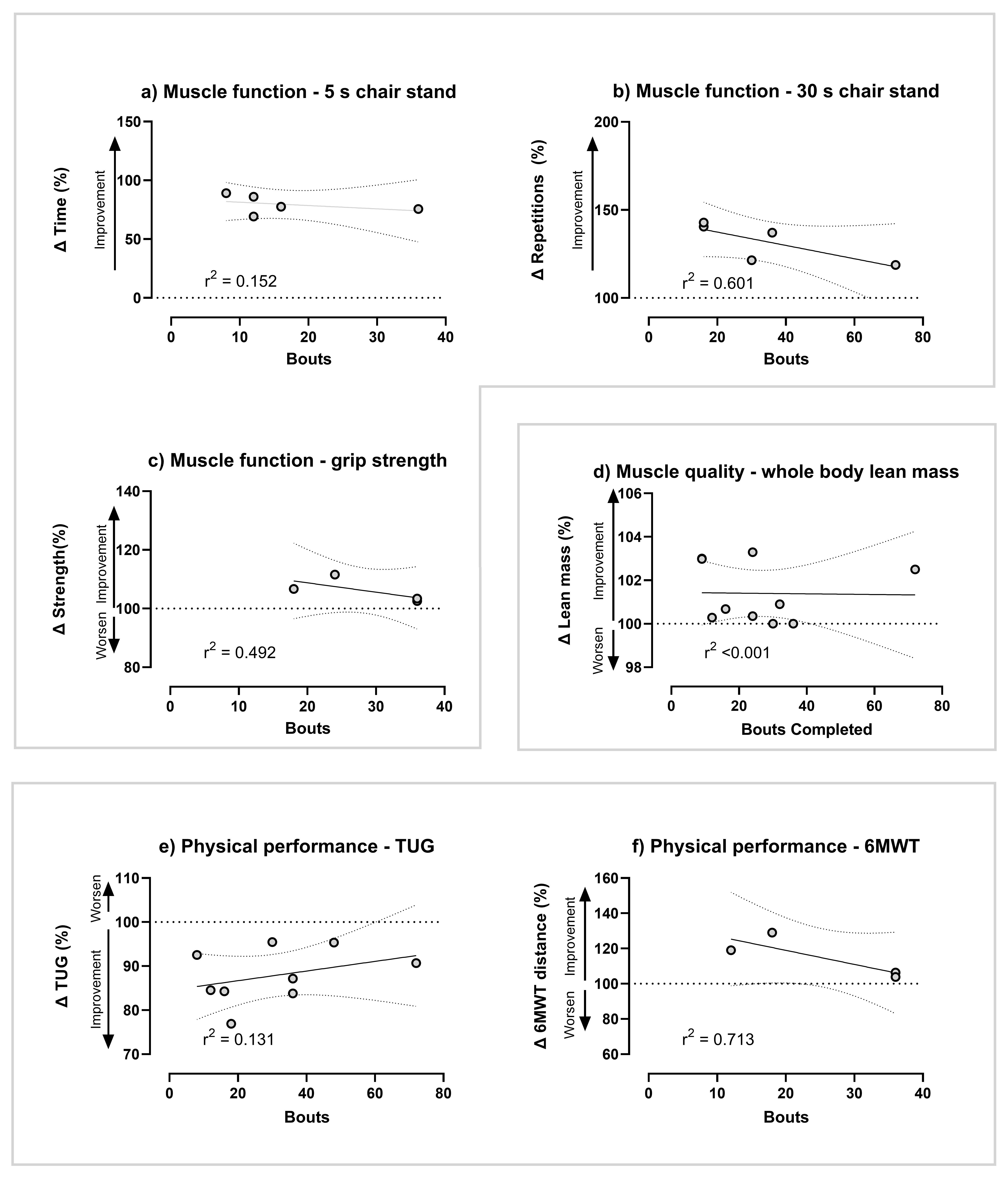

Supplement: Supplementary Figure 1 — Percent change (Δ%) in outcome measures for muscle function (a) 5 s chair stand, (b) 30 s chair stand, (c) grip strength, muss quality (d) lean mass, or muscle performance (e) timed up and go (TUG), and (f) 6 min walk test (6MWT), all as a function of the number of bouts completed. Dashed lines indicate 95% confidence intervals. [file Image_1.JPEG]
