# Supplementary material for: High Intensity Interval Training (HIIT) as a Potential Countermeasure for Phenotypic Characteristics of Sarcopenia: A Scoping Review
Source: Front Physiol. 2021 Aug 24;12:715044. doi: 10.3389/fphys.2021.715044 (PMC8423251; doi:10.3389/fphys.2021.715044)
Supplement: Supplementary file 2 [file Table_1.DOCX]

**Supplementary material 1: Full search strategy**

Databases searched:

1. Medline
2. EMBASE
3. Web of Science
4. Cochrane Central Register of Controlled Trials (CENTRAL)
5. Scopus

**Search Terms**

**1.0 MEDLINE & 2.0 EMBASE**

| **Type** | **Terms** | **Searches** |
| --- | --- | --- |
| **Intervention** | Mesh terms | high intensity intermittent training.mp. or repeated sprint training.mp. or sprint interval training.mp. or exp High-Intensity Interval Training/ |
|  | Free-text | (“high intensity interval training” or “sprint interval training” or “HIIT” or “repeated sprint training” or “high intensity intermittent training”).tw |
| **Outcome** | Mesh terms | exp Muscle Weakness/ or sarcopenia.mp. or exp Muscle, Skeletal/ or exp SARCOPENIA/ or exp Muscular Atrophy/ |
|  | Free-text | ("muscle mass" or " muscle strength" or " grip strength" or " walking speed" or " gait speed" or " appendicular lean mass" or " skeletal muscle index" or " physical performance" or " Timed Up-and-Go test" or " muscle wasting" or "age-related muscle loss" or " myopenia" or " dynapenia" or " sarcopenia" or "sarcopenic").tw. |
| **Population** | Mesh terms | exp Aged/ or exp Middle Aged/ |
|  | Free-text | ("older adult" or elderly).tw. |

| **** | [**# ▲**](http://ovidsp.tx.ovid.com/sp-3.30.0b/ovidweb.cgi?&S=IHMEFPLFHMDDDNDONCEKOHDCJAGIAA00&Sort+Sets=descending) | **Searches** | **Results** | |
| --- | --- | --- | --- | --- |
|  |  |  | **Medline** | **EMBASE** |
|  | 1 | exp Muscle Weakness/ or sarcopenia.mp. or exp Muscle, Skeletal/ or exp SARCOPENIA/ or exp Muscular Atrophy/ | 279958 | 657202 |
|  | 2 | ("muscle mass" or " muscle strength" or " grip strength" or " walking speed" or " gait speed" or " appendicular lean mass" or " skeletal muscle index" or " physical performance" or " Timed Up-and-Go test" or " muscle wasting" or "age-related muscle loss" or " myopenia" or " dynapenia" or " sarcopenia" or "sarcopenic").tw. | 66235 | 96541 |
|  | 3 | 1 or 2 | 323627 | 711533 |
|  | 4 | exp Aged/ or exp Middle Aged/ | 4971580 | 3765003 |
|  | 5 | ("older adult" or elderly).tw. | 241304 | 343466 |
|  | 6 | 4 or 5 | 5026318 | 3838821 |
|  | 7 | high intensity intermittent training.mp. or repeated sprint training.mp. or sprint interval training.mp. or exp High-Intensity Interval Training/ | 1182 | 2250 |
|  | 8 | (high intensity interval training or sprint interval training or HIIT or repeated sprint training or high intensity intermittent training).tw. | 1771 | 2309 |
|  | 9 | 7 or 8 | 2086 | 2928 |
|  | 10 | 3 and 6 and 9 | 83 | 109 |
|  | 11 | limit 10 to (english language and humans) | 81 | 104 |

**3.0 Web of Science “Web of Science Core Collection”**

| **Set** | **Results** | **Save History / Create Alert Open Saved History** |
| --- | --- | --- |
| # 4 | 14 | \|  \| #3 AND #1  *Indexes=SCI-EXPANDED, SSCI, A&HCI, CPCI-S, CPCI-SSH, ESCI Timespan=All years* \| \| --- \| --- \| |
| # 3 | [**1,552**](http://apps.webofknowledge.com/summary.do?product=WOS&doc=1&qid=7&SID=E5xlou7e1pC5qOIk1GO&search_mode=GeneralSearch&update_back2search_link_param=yes) | **TOPIC:** (((((high intensity intermittent training.mp.) OR repeated sprint training.mp.) OR sprint interval training.mp.) OR exp High-Intensity Interval Training/)) *OR* **TITLE:** ((((((("high intensity interval training") OR "sprint interval training") OR "HIIT") OR "repeated sprint training") OR "high intensity intermittent training")))  *Indexes=SCI-EXPANDED, SSCI, A&HCI, CPCI-S, CPCI-SSH, ESCI Timespan=All years* |
| # 2 | [**141,788**](http://apps.webofknowledge.com/summary.do?product=WOS&doc=1&qid=6&SID=E5xlou7e1pC5qOIk1GO&search_mode=GeneralSearch&update_back2search_link_param=yes) | **TOPIC:** ((exp Aged/ or exp Middle Aged/)) *OR* **TITLE:** (("older adult" or elderly))  *Indexes=SCI-EXPANDED, SSCI, A&HCI, CPCI-S, CPCI-SSH, ESCI Timespan=All years* |
| # 1 | 20,748 | (**TOPIC:** ((((exp Muscle Weakness OR sarcopenia.mp.) OR exp Muscle, Skeletal) OR exp SARCOPENIA) OR exp Muscular Atrophy) *OR* **TITLE:** ((((((((((((((("muscle mass" OR " muscle strength") OR " grip strength") OR " walking speed") OR " gait speed") OR " appendicular lean mass") OR " skeletal muscle index") OR " physical performance") OR " Timed Up-and-Go test") OR " muscle wasting") OR "age-related muscle loss") OR " myopenia") OR " dynapenic") OR " sarcopenia") OR "sarcopenic"))  *Indexes=SCI-EXPANDED, SSCI, A&HCI, CPCI-S, CPCI-SSH, ESCI Timespan=All years* |

**4.0 Cochrane Central Register of Controlled Trials** (**CENTRAL)**

| **ID** | **Search** | **Results** |
| --- | --- | --- |
| #1 | MeSH descriptor: [High-intensity interval training] explode all trees | 345 |
| #2 | ((high intensity interval training or sprint interval training or HIIT or repeated sprint training or high intensity intermittent training)):ti,ab,kw | 2936 |
| #3 | #1 or #2 | 255970 |
| #4 | MeSH descriptor: [Muscle Weakness] explode all trees | 491 |
| #5 | MeSH descriptor: [Muscle, Skeletal] explode all trees | 11209 |
| #6 | MeSH descriptor: [Sarcopenia] explode all trees | 407 |
| #7 | muscle mass or "muscle strength" or "grip strength" or "walking speed" or "gait speed" or "appendicular lean mass" or "skeletal muscle index" or "physical performance" or "Timed Up-and-Go test" or "muscle wasting" or "age-related muscle loss" or "myopenia" or "dynapenia" or "sarcopeni$":ti,ab,kw (Word variations have been searched) | 29,011 |
| #8 | #4 or #5 or #6 or #7 | 36,491 |
| #9 | MeSH descriptor: [Aged, 80 and over] explode all trees | 156 |
| #10 | MeSH descriptor: [Aged] explode all trees | 1312 |
| #11 | MeSH descriptor: [Adult] explode all trees | 3494 |
| #12 | "older adults" or "aged 65 and over" or "elderly" | 57841 |
| #13 | #9 or #10 or #11 or #12 | 60,092 |
| #14 | #3 and #8 and #13 | 914 |

**5.0 Scopus**

| # | Search Terms | Results |
| --- | --- | --- |
| 10 | ( ( TITLE-ABS-KEY ( exp AND muscle AND weakness/ OR sarcopenia.mp. OR exp AND muscle, AND skeletal/ OR exp AND sarcopenia/ OR exp AND muscular AND atrophy/ ) ) OR ( ( TITLE-ABS-KEY ( muscle AND mass ) OR TITLE-ABS-KEY ( muscle AND strength ) OR TITLE-ABS-KEY ( grip AND strength ) OR TITLE-ABS-KEY ( walking AND speed ) OR TITLE-ABS-KEY ( gait AND speed ) OR TITLE-ABS-KEY ( appendicular AND lean AND mass ) OR TITLE-ABS-KEY ( skeletal AND muscle AND index ) OR TITLE-ABS-KEY ( physical AND performance ) OR TITLE-ABS-KEY ( timed AND up-and-go AND test ) OR TITLE-ABS-KEY ( muscle AND wasting ) OR TITLE-ABS-KEY ( age-related AND muscle AND loss ) OR TITLE-ABS-KEY ( myopenia ) OR TITLE-ABS-KEY ( dynapenia ) OR TITLE-ABS-KEY ( sarcopenia ) OR TITLE-ABS-KEY ( sarcopenic ) ) ) ) AND ( ( TITLE-ABS-KEY ( exp AND aged/ OR exp AND middle AND aged/ ) ) OR ( ( TITLE-ABS-KEY ( older AND adult ) OR TITLE-ABS-KEY ( elderly ) ) ) ) AND ( ( TITLE-ABS-KEY ( high AND intensity AND intermittent AND training.mp. OR repeated AND sprint AND training.mp. OR sprint AND interval AND training.mp. OR exp AND high-intensity AND interval AND training/ ) ) OR ( ( TITLE-ABS-KEY ( high AND intensity AND interval AND training ) OR TITLE-ABS-KEY ( sprint AND interval AND training ) OR TITLE-ABS-KEY ( hiit ) OR TITLE-ABS-KEY ( repeated AND sprint AND training ) OR TITLE-ABS-KEY ( high AND intensity AND intermittent AND training ) ) ) ) | 154 |
| 9 | ( TITLE-ABS-KEY ( high  AND intensity  AND intermittent  AND training.mp.  OR  repeated  AND sprint  AND training.mp.  OR  sprint  AND interval  AND training.mp.  OR  exp  AND high-intensity  AND interval  AND training/ ) )  OR  ( ( TITLE-ABS-KEY ( high  AND intensity  AND interval  AND training )  OR  TITLE-ABS-KEY ( sprint  AND interval  AND training )  OR  TITLE-ABS-KEY ( hiit )  OR  TITLE-ABS-KEY ( repeated  AND sprint  AND training )  OR  TITLE-ABS-KEY ( high  AND intensity  AND intermittent  AND training ) ) ) | 5373 |
| 8 | ( TITLE-ABS-KEY ( high  AND intensity  AND interval  AND training )  OR  TITLE-ABS-KEY ( sprint  AND interval  AND training )  OR  TITLE-ABS-KEY ( hiit )  OR  TITLE-ABS-KEY ( repeated  AND sprint  AND training )  OR  TITLE-ABS-KEY ( high  AND intensity  AND intermittent  AND training ) ) | 5373 |
| 7 | TITLE-ABS-KEY ( high  AND intensity  AND intermittent  AND training.mp.  OR  repeated  AND sprint  AND training.mp.  OR  sprint  AND interval  AND training.mp.  OR  exp  AND high-intensity  AND interval  AND training/ ) | 0 |
| 6 | ( TITLE-ABS-KEY ( exp  AND aged/  OR  exp  AND middle  AND aged/ ) )  OR  ( ( TITLE-ABS-KEY ( older  AND adult )  OR  TITLE-ABS-KEY ( elderly ) ) ) | 1384566 |
| 5 | ( TITLE-ABS-KEY ( older  AND adult )  OR  TITLE-ABS-KEY ( elderly ) ) | 1,383,635 |
| 4 | TITLE-ABS-KEY ( exp  AND aged/  OR  exp  AND middle  AND aged/ ) | 1,124 |
| 3 | ( TITLE-ABS-KEY ( exp  AND muscle  AND weakness/  OR  sarcopenia.mp.  OR  exp  AND muscle,  AND skeletal/  OR  exp  AND sarcopenia/  OR  exp  AND muscular  AND atrophy/ ) )  OR  ( ( TITLE-ABS-KEY ( muscle  AND mass )  OR  TITLE-ABS-KEY ( muscle  AND strength )  OR  TITLE-ABS-KEY ( grip  AND strength )  OR  TITLE-ABS-KEY ( walking  AND speed )  OR  TITLE-ABS-KEY ( gait  AND speed )  OR  TITLE-ABS-KEY ( appendicular  AND lean  AND mass )  OR  TITLE-ABS-KEY ( skeletal  AND muscle  AND index )  OR  TITLE-ABS-KEY ( physical  AND performance )  OR  TITLE-ABS-KEY ( timed  AND up-and-go  AND test )  OR  TITLE-ABS-KEY ( muscle  AND wasting )  OR  TITLE-ABS-KEY ( age-related  AND muscle  AND loss )  OR  TITLE-ABS-KEY ( myopenia )  OR  TITLE-ABS-KEY ( dynapenia )  OR  TITLE-ABS-KEY ( sarcopenia )  OR  TITLE-ABS-KEY ( sarcopenic ) ) ) | 501,096 |
| 2 | ( TITLE-ABS-KEY ( muscle  AND mass )  OR  TITLE-ABS-KEY ( muscle  AND strength )  OR  TITLE-ABS-KEY ( grip  AND strength )  OR  TITLE-ABS-KEY ( walking  AND speed )  OR  TITLE-ABS-KEY ( gait  AND speed )  OR  TITLE-ABS-KEY ( appendicular  AND lean  AND mass )  OR  TITLE-ABS-KEY ( skeletal  AND muscle  AND index )  OR  TITLE-ABS-KEY ( physical  AND performance )  OR  TITLE-ABS-KEY ( timed  AND up-and-go  AND test )  OR  TITLE-ABS-KEY ( muscle  AND wasting )  OR  TITLE-ABS-KEY ( age-related  AND muscle  AND loss )  OR  TITLE-ABS-KEY ( myopenia )  OR  TITLE-ABS-KEY ( dynapenia )  OR  TITLE-ABS-KEY ( sarcopenia )  OR  TITLE-ABS-KEY ( sarcopenic ) ) | 501,094 |
| 1 | TITLE-ABS-KEY ( exp  AND muscle  AND weakness/  OR  sarcopenia.mp.  OR  exp  AND muscle,  AND skeletal/  OR  exp  AND sarcopenia/  OR  exp  AND muscular  AND atrophy/ ) | 3 |
